# Depth differentiation of microbial communities and nutrient cycling functional genes in semi-arid riparian soil

**DOI:** 10.3389/fmicb.2025.1717707

**Published:** 2025-12-18

**Authors:** Yutong Liu, Jinxuan Wang, Wei Wei, Manhong Xia, Deshuai Ji, Fan Wang, Xuanming Zhang, Wenke Wang

**Affiliations:** 1 School of Water and Environment, Chang’an University, Xi’an, Shaanxi, China; 2 Key Laboratory of Subsurface Hydrology and Ecological Effect in Arid Region of the Ministry of Education, Chang’an University, Xi’an, Shaanxi, China; 3 Key Laboratory of Eco-hydrology and Water Security in Arid and Semi-arid Regions of Ministry of Water Resources, Chang’an University, Xi’an, Shaanxi, China; 4 School of Architecture, Chang’an University, Xi’an, Shaanxi, China

**Keywords:** microbial community, metagenomic, nutrient cycling, functional gene, semi-arid riparian soil

## Abstract

**Introduction:**

Microbial communities and their associated carbon, nitrogen, and phosphorus metabolic processes play a role in maintaining ecological functions and nutrient cycling in riparian zones. However, systematic research on the coupling mechanisms of carbon, nitrogen, and phosphorus biogeochemical processes in soil profiles of semi-arid riparian soil is still limited.

**Methods:**

This study focused on the riparian zone of the Tuwei River, a typical semi-arid river. Metagenomic sequencing was used to analyze the composition of microbial communities and their carbon, nitrogen, and phosphorus metabolic functions across different soil depths along the river.

**Results:**

The dominant taxa across all depths and river sections were *Proteobacteria* (average relative abundance 49.85%) and *Serratia* (11.23%). Results from ANOVA and Tukey–Kramer post-hoc multiple comparison tests showed that microbial diversity significantly decreased with increasing soil depth (*p* < 0.05). Gene families associated with carbon fixation (*accC, pccB*), denitrification (*nosZ, nirK*), and phosphorus metabolism (*purC, guaB, pyrG*) were significantly enriched in surface soils and showed clear depth-dependent declines (*p* < 0.05). Partial Mantel tests revealed that microbial metabolic functions were significantly correlated with porosity (*p* < 0.05), soil organic carbon, total nitrogen, and total phosphorus, confirming that nutrient availability and soil structure are key regulators of microbial biogeochemical functions.

**Conclusion:**

Our findings reveal that nutrient availability and soil structure jointly regulate the vertical distribution of microbial metabolic functions. These insights provide a scientific basis for ecological restoration and soil management in semi-arid riparian zones, where optimizing surface structure and nutrient inputs can stimulate microbial-driven biogeochemical cycling. Key functional taxa and genes may also serve as sensitive indicators for evaluating restoration effectiveness under climate-induced stress.

## 1 Introduction

Riparian zones, as the interface between terrestrial and aquatic ecosystems, play a crucial role in buffering, filtering, pollution purification, and hydrological regulation. The ecological integrity of these zones is essential for maintaining watershed ecological safety and biogeochemical cycling (Yang et al., 2023; Ye et al., 2024). In this system, soil microorganisms are key biological factors that drive processes such as organic matter decomposition, nutrient transformation, and pollutant removal (Philippot et al., 2024; Stokes et al., 2014), directly regulating the stability of riparian ecosystem functions (Li et al., 2025; Zhao and Wu, 2025). This role is particularly pronounced in semi-arid regions, where microorganisms play an irreplaceable role in maintaining soil quality, supporting vegetation recovery, and enhancing system resilience due to limited water and nutrient resources (Alori et al., 2024; Zheng et al., 2024). They are key indicators for assessing the ecological health and service functions of riparian zones (Wen et al., 2023). Therefore, studying the composition characteristics and ecological functions of soil microbial communities in riparian zones of semi-arid regions is of great scientific significance for developing regional ecological management strategies.

Soil microbial-driven carbon (C), nitrogen (N), and phosphorus (P) cycles are core processes in regulating nutrient dynamics in riparian zones (Wang L. L. et al., 2022). A systematic analysis of microbial-mediated carbon, nitrogen, and phosphorus metabolic functions and their potential is crucial for understanding the biological control mechanisms of nutrient cycling (Luo et al., 2025). Microorganisms directly control soil nutrient availability and ecosystem productivity through metabolic pathways such as carbon fixation, carbon degradation, organic degradation and synthesis, denitrification, purine metabolism, and pyrimidine metabolism (Liu Z. et al., 2024). Therefore, systematically analyzing the functional potential of microbial communities is key to revealing the mechanisms of element cycling regulation in riparian zones. Metagenomics, with its ability to analyze functional genes directly without the need for cultivation (Hu et al., 2025), provides a comprehensive approach to uncovering microbial metabolic networks and their responses to environmental factors, making it a powerful tool for studying riparian ecosystem functions (Kang et al., 2024; Liu et al., 2025).

In riparian ecosystem studies, understanding the structural composition and metabolic functional characteristics of riparian zone microorganisms is crucial for comprehending soil ecological processes and material cycling (Yang et al., 2023). Previous studies have shown that soil microorganisms in riparian zones play a central role in the cycling of key nutrients such as carbon, nitrogen, and phosphorus (Wang et al., 2020). In regions with high disturbance or significant environmental stress, the stability and adaptability of their ecological functions are more indicative (Cui et al., 2018). For example, in semi-arid riparian ecosystems, both plant-derived and microbial-derived organic carbon jointly determine soil carbon accumulation, with microorganisms playing a key role in carbon fixation and organic matter transformation (Liu et al., 2025). However, in watersheds with high agricultural development or land use intensity, a decline in soil organic carbon (SOC) levels leads to the disruption of microbial community structure and weakened functional gene expression, thereby inhibiting the efficiency of carbon and nitrogen cycling (Kang et al., 2024; Li W. J. et al., 2022). Additionally, soil microorganisms in riparian zones are highly sensitive to changes in land cover types, with different vegetation patterns significantly altering microbial community composition and metabolic potential (Zhao and Wu, 2025). In headwater rivers of humid regions, reduced vegetation cover leads to decreased organic matter input, thus affecting the expression levels of key functional genes involved in carbon fixation and denitrification (Wang et al., 2020). Furthermore, in agricultural and semi-natural riparian zones, studies have found a strong coupling between microbial nitrogen cycling pathways, moisture conditions, and anthropogenic disturbances, with functional genes exhibiting high spatial heterogeneity in different ecological niches (Xin et al., 2023; Zhang et al., 2021). In riparian soils of boreal forest regions, the moisture gradient significantly affects bacterial and fungal communities and decomposition metabolic activity, suggesting that microbial processes have regional adaptability in regulating organic matter degradation and nutrient release (Annala et al., 2022; Zhu et al., 2025).

Compared to riparian ecosystems in humid and temperate regions, research on microbial functional properties in riparian zones of arid and semi-arid areas is significantly lacking. Semi-arid ecosystems face unique environmental pressures such as water scarcity, strong nutrient heterogeneity, and unstable soil structures (Arce et al., 2023; Jiao et al., 2022). Microbial communities in these areas may evolve specialized composition strategies and functional adaptation mechanisms (Hu et al., 2021). Currently, little is known about the following key scientific questions: how do microbial communities in semi-arid riparian zones distribute along soil profiles and river gradients, how do their carbon, nitrogen, and phosphorus metabolic potentials respond to gradients of water, nutrients, and soil structure, and which environmental factors mainly drive these functional differences?

As a typical semi-arid river, the Tuwei River is located in the transition zone between the Loess Plateau and the desert margin, where its riparian ecosystem is severely affected by soil erosion, vegetation degradation, and water stress, resulting in significant ecological vulnerability (Yang et al., 2016). Based on this, this study focuses on the Tuwei River, a representative river in the semi-arid region of the Loess Plateau. We established profiles along the riparian zone for stratified sampling and applied metagenomic sequencing technology. The objectives of this study are to: (1) reveal the spatial variation in the composition and diversity of soil microbial communities in the riparian zone; (2) elucidate the distribution patterns and metabolic potential of microbial carbon, nitrogen, and phosphorus metabolic genes; (3) identify key environmental driving factors influencing microbial community structure and functional gene composition.

Therefore, in accordance with the objectives of this study, we propose the following scientific hypotheses: (1) The composition and diversity of soil microbial communities in semi-arid riparian zones exhibit significant spatial heterogeneity, characterized by a marked decline in microbial diversity with increasing soil depth; (2) Microbial functional genes involved in carbon, nitrogen, and phosphorus metabolism display distinct vertical distribution patterns, with surface soils (0–20 cm) exhibiting higher metabolic potential; (3) The vertical differentiation of microbial community structure and functional gene composition is primarily regulated by environmental factors, with porosity and soil nutrient availability identified as the main driving variables. This study will enhance the understanding of the nutrient cycling mechanisms driven by microorganisms in riparian zones of semi-arid regions and provide theoretical support for regional soil and water conservation and ecological restoration.

## 2 Materials and methods

### 2.1 Study region

This study selected the Tuwei River Basin as the research area (38°00′–38°50′N, 110°10′–110°40′E). The Tuwei River is an important first-order tributary of the Yellow River, located on the northern edge of the Loess Plateau in Shaanxi Province, China. It is a typical inland semi-arid river basin (Supplementary Figure S1). The average annual precipitation in the basin is about 400 mm, while the annual evaporation exceeds 2000 mm, leading to severe soil moisture deficits, difficulty in vegetation recovery, and significant ecosystem vulnerability.

The landscape features along the Tuwei River are distinct. The upper reach has a relatively flat terrain with a wide riverbed and slow flow. The middle reach exhibits a pronounced V-shaped valley morphology, with steeper terrain, a narrower riverbed, and faster flow, resulting in significant soil erosion. The lower reach is characterized by a broad alluvial plain where the river occasionally overflows, and the soil has higher salinity with relatively slower water flow. The natural vegetation along the riverbank is dominated by drought resistant shrubs and herbaceous plants, with low vegetation cover and poor ecosystem stability.

### 2.2 Soil sampling and processing

In August 2024, soil samples were collected from several sampling points located in the upstream, midstream, and downstream riparian zones of the Tuwei River (Supplementary Table S1). At each sampling point, the horizontal distance from the river, the width of the riparian zone, vegetation cover, and land use type were carefully considered and controlled to ensure consistency. Each sampling point was located at a horizontal distance of 20–50 meters from the river, which corresponds to the typical transition zone in riparian ecosystems. This range effectively captures the combined influence of fluvial hydrological processes and adjacent terrestrial factors, ensuring both the representativeness of the sampling area and its ecological responsiveness to riverine impacts. A riparian zone width of 100 meters was adopted based on standard estimations for such ecosystems, allowing for broad representativeness and adequate reflection of microbial community variations across different river sections. In addition, all sampling points shared consistent vegetation types and coverage, with drought-tolerant herbaceous plants and shrubs selected to match the local ecological conditions. All sampling points were located in areas with similar land use types to avoid the influence of land use differences on the soil microbial community and metabolic functions.

At each sampling point, soil samples were collected from four depth intervals (0–20 cm, 20–40 cm, 40–60 cm, and 60–80 cm), with three independent biological replicates per layer. Due to the shallow groundwater level and limited soil depth in the downstream zone, sampling at 60–80 cm was not feasible in that section, resulting in a total of 33 valid soil samples. The specific sampling information is provided in Supplementary Table S1. A sterile shovel was used during sampling to avoid external contamination. The collected soil samples were kept in dark conditions and transported to the laboratory as soon as possible. In the lab, all soil samples were first passed through a 2 mm mesh sieve to remove plant roots, stones, and other debris. Each sample was then divided into two parts: one part was used to measure soil physicochemical properties, and the other was placed into sterile 50 mL centrifuge tubes and stored at temperatures below −80 °C for subsequent genomic DNA extraction and molecular biology analysis.

### 2.3 Soil properties analyses

The soil physicochemical properties measured included soil moisture content, soil bulk density, porosity, particle size, pH, electrical conductivity (EC), soil organic carbon (SOC), dissolved organic carbon (DOC), total nitrogen (TN), ${\mathrm{NO}}_{3}^{-}-N,$ ${\mathrm{NO}}_{2}^{-}-N,$ ${\mathrm{NH}}_{4}^{+}-N,$ and total phosphorus (TP).

Soil moisture content was measured *in situ* using a handheld moisture sensor (MQ-SCTR, China). Soil bulk density was determined using the core method, with wet soil samples dried at 105 °C for 24 h to calculate the density. By fully saturating the soil sample with water and measuring the amount of water it can hold, the porosity of the soil is calculated by dividing the pore volume by the total volume of the soil sample. Particle size composition was measured using a laser particle size analyzer (LT 2200E, China). Soil pH was measured using an electrode method. EC was determined by a 1:5 (m/V) water extraction, with the sample shaken at 20 °C and the conductivity of the supernatant measured at 25 °C. SOC was measured using the potassium dichromate oxidation method with external heating (Walkley and Black, 1934), while DOC was determined after water extraction, centrifugation, and filtration through a 0.45 μm filter, using a TOC analyzer (TOC-L CPH, Japan) (Sugimura and Suzuki, 1988). TN was measured using the Kjeldahl method (Sáez-Plaza et al., 2013), with digestion in a graphite digestion instrument (SH220F, China) followed by distillation and titration with a Kjeldahl distillation unit (K9840, China). ${\mathrm{NO}}_{3}^{-}-N$ and ${\mathrm{NO}}_{2}^{-}-N$ were extracted using 1 mol/L potassium chloride and determined by dual wavelength colorimetry (Norman et al., 1985), while ${\mathrm{NH}}_{4}^{+}-N$ was measured using the indophenol blue colorimetric method (Selmer-Olsen, 1971). TP was determined by the NaOH fusion-molybdenum antimony colorimetric method (MD SpectraMax190, United States) (Pulliainen and Wallin, 1996).

### 2.4 DNA extraction, metagenomic sequencing and functional analysis

To analyze the functional composition and metabolic potential of microbial communities at different soil depths, this study performed total DNA extraction, metagenomic library construction, sequencing, and functional annotation analysis on soil samples from each sampling depth. Total DNA from the soil was extracted using the E. Z. N. A.^®^ Stool DNA Kit (Omega Bio-tek, Norcross, GA, United States), following the manufacturer’s protocol to ensure extraction efficiency and DNA integrity. The quality of the extracted DNA was assessed using both a NanoDrop 2000 spectrophotometer and a Qubit 4.0 fluorometer (Thermo Fisher Scientific, United States) for dual quality control. DNA samples meeting the quality standards were selected for sequencing library construction.

DNA samples that met the quality standards were subjected to paired-end metagenomic sequencing (PE150 mode) on the Illumina platform. Library construction was performed using the TruSeq DNA Sample Preparation Kit (Illumina, FC-121-2001). All libraries were quantified using a high-sensitivity double-stranded DNA assay on the Qubit system, and fragment size and quality distribution were verified using the Agilent 2,100 Bioanalyzer. The raw sequencing data (Raw Reads) were first filtered for adapter sequences and low quality bases using Trimmomatic software^1^ (Bolger et al., 2014). Specifically, adapter removal was performed with the ILLUMINACLIP module (adapters. fa:2:30:10). For quality trimming, the SLIDINGWINDOW option was applied (window size = 4, average quality threshold = 15). Bases with quality scores <20 at the 3′ end were trimmed, and reads containing any bases with scores <10 after trimming were discarded. Reads with more than 10% ambiguous bases (N content) or shorter than 75 bp were also removed. These criteria ensured that only high-quality sequences were retained for downstream analysis. To remove host contamination sequences, the cleaned reads were aligned to the human genome (NCBI) using BWA mem (parameters: -M -k32 -t16, http://bio-bwa.sourceforge.net/bwa.shtml). The alignment results were used to filter out host-related sequences, and the resulting high-quality non-host sequences (Clean Reads) were used for subsequent analysis.

The assembly process was performed using MEGAHIT (v1.2.9) for deduplication and contig assembly, with a minimum contig length set to 500 bp (parameters: -min-contig-len 500) (Li et al., 2015). Open reading frames (ORFs) were predicted using Prodigal software (v2.6.3) in “meta” mode to generate protein-coding gene information. To construct a non-redundant gene catalog with redundancy removed, all ORFs were clustered using CD-HIT (v4.8.1, parameters: -n9 -c0.95 -G0 -M0 -d0 -aS0.9 -r1) (Fu et al., 2012), with the longest sequence representing the representative gene of each cluster. Clean Reads from each sample were then aligned to the representative gene catalog, and gene abundance (TPM, transcripts per million) was calculated using Salmon (v1.4.0) software to normalize for biases introduced by gene length and sequencing depth (Patro et al., 2017). Finally, the gene abundance was calculated using following formulas:


$$\mathrm{TPM}=\frac{N_{g}}{L_{g}}\times \frac{1}{\sum j\frac{N_{j}}{L_{j}}}\times 10^{6}$$

*N_g_*, the average number of reads mapped to the *g* gene; *L_g_*, the number of nucleotides in the *g* gene; The index *j* stands for the set of all genes determined in a catalog, and *g* is an index indicating a particular gene (Xie et al., 2021).

All non-redundant gene sequences were first translated into amino acid sequences and then subjected to annotation against multiple public databases using DIAMOND BLASTP (v2.0.13) to identify gene functions. The databases used for alignment were the eggNOG database,^2^ NR database, KEGG database,^3^ CCycDB database,^4^ NCycle database,^5^ and PCycle database.^6^ The alignment parameters were set to E-value ≤ 0.00001 and sequence identity ≥ 70%. KEGG homologous annotations were performed using KofamScan^7^ and the HMMSEARCH package.^8^

### 2.5 Bioinformatics and statistical analysis

To assess the diversity characteristics of microbial communities in the semi-arid riparian zone, the sequencing data were first rarefied using Mothur software (version 1.45.1) paper (Schloss et al., 2009), and alpha diversity indices such as species richness and the Shannon diversity index were calculated. For beta diversity analysis, the “vegan” package (version 2.6–4) (Oksanen, 2025) in R software (version 4.4.3) was employed to construct Bray-Curtis distance matrices with 999 permutations. The microbial community similarity was analyzed using Spearman’s rank correlation coefficient (r > 0.6) to test the relationships between community structure and functional genes related to carbon, nitrogen, and phosphorus metabolism paper.

To eliminate the effects of covariance between soil physicochemical properties, the “VennDiagram” package (Chen, 2025) was applied to identify shared and unique genera among different samples. Additionally, variance inflation factor (VIF) analysis was performed with the “vegan” package (Oksanen, 2025) to retain variables with VIF values below 10 for further analysis. A community dissimilarity matrix was generated to assess the spatial variation of microbial communities (beta diversity), using Bray-Curtis distances calculated in the “vegan” package (Oksanen, 2025).

To quantify the soil nutrient availability and assess potential resource constraints on microbial metabolism, we constructed two composite indices: the Nutrient Availability Index (NAI) and the Resource Limitation Index (RLI). The NAI was developed based on z-score standardized values of six key edaphic variables (SOC, DOC, TN, ${\mathrm{NO}}_{3}^{-}-N,$ ${\mathrm{NH}}_{4}^{+}-N,$ and TP) representing carbon, nitrogen, and phosphorus resources. Specifically, carbon availability was calculated as the mean of standardized SOC and DOC values; nitrogen availability as the mean of TN, ${\mathrm{NO}}_{3}^{-}-N$ and ${\mathrm{NH}}_{4}^{+}-N;$ and phosphorus availability as the standardized TP value. The NAI was then computed as the average of the three nutrient-specific components. To reflect the intensity of nutrient limitation, we derived the RLI by applying a min-max normalization to the NAI across all samples (range 0–1) and calculating its inverse form (RLI = 1 – rescaled NAI), where higher values indicate greater resource constraints. This integrative approach enables the spatially explicit characterization of nutrient gradients and microbial habitat conditions across soil depths and river reaches. This composite index framework is conceptually aligned with prior studies employing z-score-based aggregation of nutrient-related variables to infer microbial nutrient limitations and ecological stoichiometry patterns, especially in arid and semi-arid environments (Cui et al., 2024; Wang et al., 2023b). While not intended to replace direct measurements or enzyme-based limitation assessments, these indices provide a reproducible and interpretable metric for linking soil nutrient status to microbial ecological function.

Furthermore, the partial Mantel test analysis, using the “dplyr” (Hadley Wickham, 2023) and “linkET” packages (Huang, 2021), was conducted to explore the correlations between microbial community structure, functional metabolism (C, N, P), and environmental factors. The dissimilarity matrix for environmental factors was computed using Euclidean distance. The results were visualized using the “pheatmap” package (Kolde, 2019) to create functional gene abundance heatmaps, which aided in understanding the spatial distribution of microbial metabolism across different soil depths and river gradients.

To evaluate the differences in soil physicochemical properties, microbial abundance, and functional gene abundance across soil depths, one-way analysis of variance (ANOVA) was conducted, followed by Tukey–Kramer post-hoc multiple comparison tests for significance testing paper. We assessed the normality of the model residuals using the Shapiro–Wilk test and log- or square-root transformed the data where necessary to meet the assumptions of normality and homogeneity of variances for ANOVA. For the visualization of soil physicochemical properties, diversity indices, and functional gene abundances, Origin 2021 software was used, while other figure visualizations were generated using the “ggplot2” package in R to ensure both scientific accuracy and aesthetic quality paper.

## 3 Results and discussion

### 3.1 Spatial variability of physicochemical properties in semi-arid riparian soil

**Figure 1:**
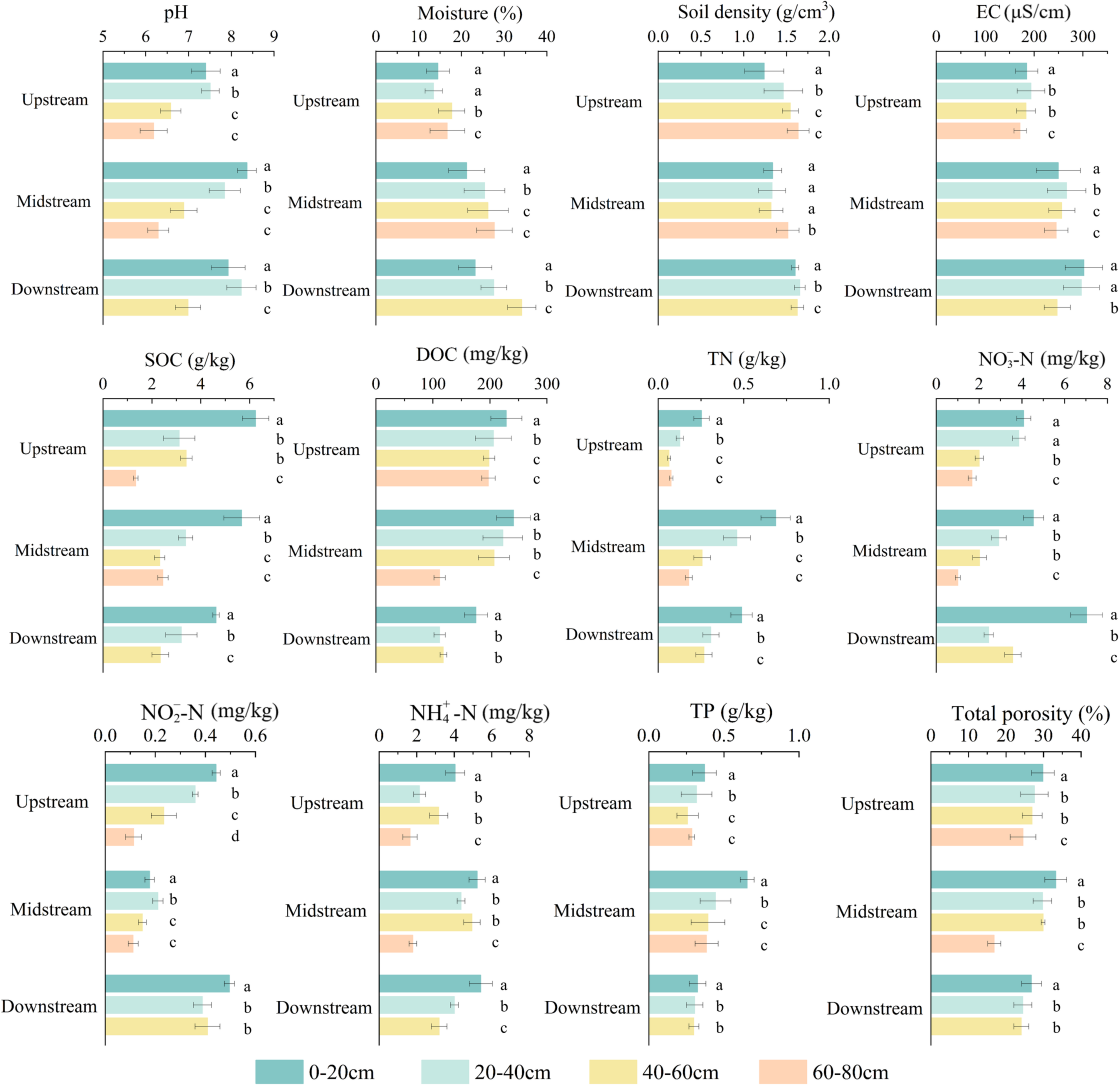
Physicochemical properties of soils at different depths in the upstream, midstream, and downstream riparian zones. Upstream: upstream riparian zone; Midstream: midstream riparian zone; Downstream: downstream riparian zone. 0–20 cm, 20–40 cm, 40–60 cm, and 60–80 cm represent soil samples from the corresponding depths. Different letters indicate significant differences, *p* < 0.05.

The changes in soil physicochemical properties at different depths along the upstream, midstream, and downstream riparian zones are shown in Figure 1. Overall, the pH, EC, SOC, DOC, TN, ${\mathrm{NO}}_{3}^{-}-N,$ ${\mathrm{NO}}_{2}^{-}-N,$ TP, and porosity indicators exhibited significant spatial variation.

Several physicochemical indicators showed notable differences along the river profile. Soil electrical conductivity (EC) was generally higher in the midstream and downstream riparian zones (204.69–340.20 μS/cm) compared to the upstream zone (162.53–207.69 μS/cm), likely due to cumulative salt deposition and hydrological concentration in these sections (Bai et al., 2023). Surface SOC (0–20 cm) decreased from upstream to downstream, while DOC was significantly higher in the upstream and midstream zones (185.63–256.07 mg/kg) than in the downstream zone (101.78–206.17 mg/kg) (*p* < 0.05), suggesting better organic matter input and retention capacity in the upper reaches (Ye et al., 2019). In contrast, TN was lowest in the upstream zone (0.05–0.21 g/kg) and higher in the midstream and downstream zones (0.20–0.77 g/kg). Moreover, ${\mathrm{NO}}_{3}^{-}-N$ concentration peaked in surface soils of the downstream riparian zone (7.49 ± 1.72 mg/kg), indicating favorable redox conditions for nitrate accumulation (Jia et al., 2024). Other indicators, such as pH and porosity, did not show statistically significant variation among river zones.

Comparing along soil depth, soil nutrients (SOC, DOC, TN, ${\mathrm{NO}}_{3}^{-}-N,$ ${\mathrm{NO}}_{2}^{-}-N,$ ${\mathrm{NH}}_{4}^{+}-N,$ TP, and porosity) were mainly concentrated in the surface soil (0–20 cm), with SOC, TN, and ${\mathrm{NO}}_{3}^{-}-N$ being particularly significant. Specifically, SOC concentrations in the surface soil (0–20 cm) in the upstream, midstream, and downstream riparian zones (4.50–6.78 g/kg) were significantly higher than in the deeper soils (60–80 cm) (1.25–2.66 g/kg) (*p* < 0.05). TN concentrations in the surface soil (0–20 cm) (0.21–0.77 g/kg) were significantly higher than in the deeper soil (60–80 cm) (0.07–0.20 g/kg) (*p* < 0.05). ${\mathrm{NO}}_{3}^{-}-N$ concentrations in the surface soil (0–20 cm) (4.09–7.77 mg/kg) were also significantly higher than in the deeper soil (60–80 cm) (0.89–1.86 mg/kg) (*p* < 0.05). This is closely related to the high organic matter content, nutrient accumulation, and microbial activity in the surface soil, while deeper soils are limited by external inputs, poor oxygen permeability, and nutrients that are easily consumed by plant roots and microorganisms (Hale et al., 2014; Zhao et al., 2020), leading to a significant decrease in SOC, TN, ${\mathrm{NO}}_{3}^{-}-N,$ and other indicators.

Statistical validation through the Tukey–Kramer multiple comparison test indicated that differences in the Nutrient Availability Index (NAI) and Resource Limitation Index (RLI) among river reaches (upstream, midstream, downstream) were minimal, suggesting overall similarity in nutrient status across the horizontal gradient. In contrast, along the vertical gradient, NAI exhibited a clear depth-dependent decline, with surface soils (0–20 cm) consistently showing higher nutrient availability than subsoils (60–80 cm). Conversely, RLI increased with depth, indicating stronger resource constraints in deeper soil layers. These results revealed significant differences between surface and deep soils (Tables 1, 2, *p* < 0.05).

**Table 1:** The nutrient availability index at different depths in the upstream, midstream, and downstream riparian zones.

River section	0–20 cm	20–40 cm	40–60 cm	60–80 cm
Upstream	−0.28 ± 0.13^a^	−2.07 ± 0.32^b^	−2.09 ± 0.02^b^	−2.86 ± 0.16^c^
Midstream	1.66 ± 0.03^a^	−0.32 ± 0.20^b^	−1.06 ± 0.24^bc^	−2.71 ± 0.06^c^
Downstream	−0.38 ± 0.01^a^	−1.96 ± 0.08^ab^	−2.40 ± 0.01^b^	

Different letters indicate significant differences, *p* < 0.05.

**Table 2:** The resource limitation index at different depths in the upstream, midstream, and downstream riparian zones.

River section	0–20 cm	20–40 cm	40–60 cm	60–80 cm
Upstream	0.43 ± 0.04^a^	0.82 ± 0.09^b^	0.83 ± 0.02^b^	0.99 ± 0.01^c^
Midstream	0^a^	0.44 ± 0.05^b^	0.60 ± 0.07^bc^	0.96 ± 0.04^c^
Downstream	0.45 ± 0.01^a^	0.80 ± 0.04^b^	0.89 ± 0.02^b^	

Different letters indicate significant differences, *p* < 0.05.

In the soil particle composition analysis, the average sand content (0.05–2 mm) was 96.76%. The soil texture ternary diagram (Supplementary Figure S2) shows that all samples are clustered within the sandy soil zone, with slight variations in clay and silt content. According to the USDA soil texture classification standards, the soils in this region are classified as sandy, characterized by good aeration but poor water retention. There were no significant differences in soil texture among the sampling sites.

### 3.2 Composition and diversity of microbial communities in semi-arid riparian soil

**Figure 2:**
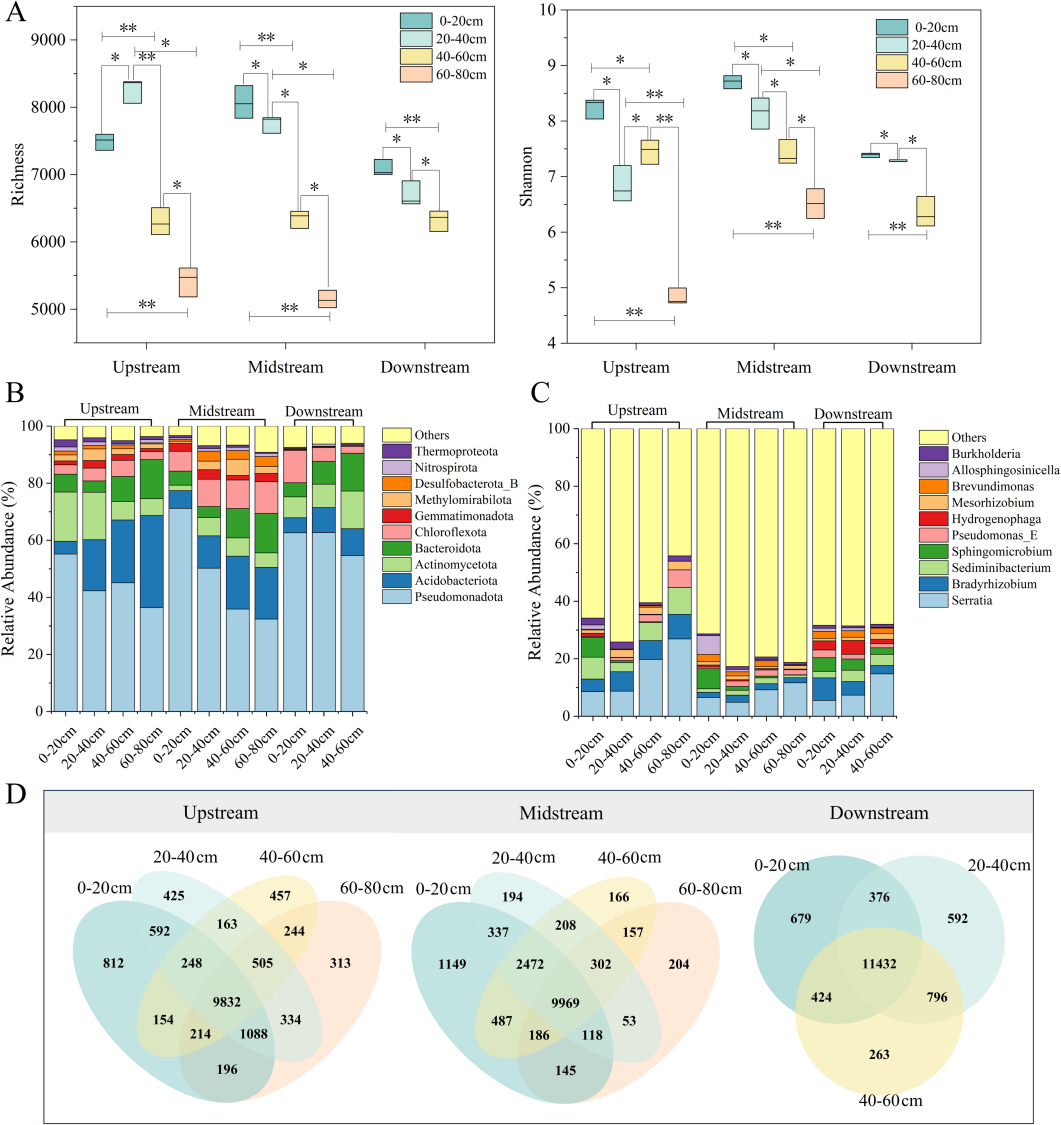
Microbial diversity and community composition at different soil depths in the upstream, midstream, and downstream riparian zones. **(A)** Alpha diversity indices of riparian zone soil microbes. Upstream: upstream riparian zone; Midstream: midstream riparian zone; Downstream: downstream riparian zone. 0–20 cm, 20–40 cm, 40–60 cm, and 60–80 cm represent soil samples from the corresponding depths. * indicates p < 0.05, ** indicates *p* < 0.01. Relative abundance of soil microbial communities at the phylum **(B)** and genus **(C)** levels. The top 10 dominant populations based on relative abundance at the phylum and genus levels are listed. **(D)** Venn diagram of microbial genera in riparian zone soils.

To systematically assess the composition and diversity of soil microbial communities in riparian zones of semi-arid regions, this study conducted preliminary analysis based on metagenomic sequencing data. The results showed that the diversity indices in the upstream and midstream riparian zones at 0–40 cm were significantly higher than those in the downstream riparian zone (*p* < 0.05). Surface soils (0–20 cm) generally exhibited higher microbial *α*-diversity, including species richness and Shannon diversity index (Figures 2A,B), with this trend being particularly significant in the midstream and downstream riparian zone soils (*p* < 0.05). This trend was closely related to the higher nutrient contents, such as SOC, TN, and ${\mathrm{NO}}_{3}^{-}-N,$ in the surface soils (Figure 1), reflecting that nutrient supply is an important foundation for maintaining microbial diversity (Wang et al., 2023a; Zheng et al., 2019). The concentrations of these indicators in shallow soils (0–20 cm) were significantly higher than in deep soils (60–80 cm) (*p* < 0.05), supporting higher microbial metabolic activity and niche differentiation in surface communities (Leewis et al., 2022). These results were consistent with the Venn diagram analysis (Figure 2D), where surface samples had the highest number of unique genera, and the number of unique genera rapidly declined with increasing depth, leading to a simplified community structure in deeper soils.

In terms of community composition, bacteria dominate the microbial communities in the riparian zone soils of the study area, accounting for more than 99% and representing an absolute dominance in all samples (Figures 2B,C). Among them, *Proteobacteria* (average relative abundance 49.85%) and *Serratia* (average relative abundance 11.23%) are the most dominant groups (Figures 2B,C), widely distributed across the upstream, midstream, and downstream riparian zones as well as different soil layers, showing strong environmental adaptability (Dixon et al., 2025). Along the river gradient, the abundance of *Chloroflexota* and *Desulfobacterota_B* in the midstream riparian zone was significantly higher than in the upstream and downstream zones (Figure 2B) (*p* < 0.05). Combined with the midstream’s moderate soil electrical conductivity and higher organic matter concentration, it is inferred that these groups have an ecological niche advantage in this region, reflecting the sensitivity of specific microbial groups to hydrological and soil conditions (Williams et al., 2024). *Bradyrhizobium* and *Sediminibacterium* exhibited the lowest abundance in the midstream (Figure 2C), possibly due to nutrient competition or niche overlap being limited by local environmental conditions (Galanova et al., 2025). Along the soil depth gradient, the relative abundance of *Proteobacteria* showed a significant decreasing trend in the vertical profile, decreasing with soil depth (*p* < 0.05) (Figure 2B; Supplementary Figure S3), indicating their preference for surface environments with higher oxygen content and organic matter (Shao et al., 2021). On the other hand, *Serratia* and *Bacteroidota* showed a significant increase with depth (*p* < 0.05) (Figure 2B; Supplementary Figure S3), which may reflect their metabolic advantage in anaerobic or low carbon source environments (Kang et al., 2021). In grassland restoration studies, communities shift from oligotrophic to eutrophic types as vegetation recovers and soil nutrients increase (Liao et al., 2023). In this study, although some soil layers in the riparian zone exhibit eutrophic characteristics, a relatively high proportion of oligotrophic groups remain in the deeper soils, demonstrating adaptive differences along the nutrient gradient.

### 3.3 Vertical stratification of functional genes involved in carbon nitrogen and phosphorus metabolism

#### 3.3.1 Carbon metabolism pathways and key gene distributions showing surface dominance and deep layer attenuation

**Figure 3:**
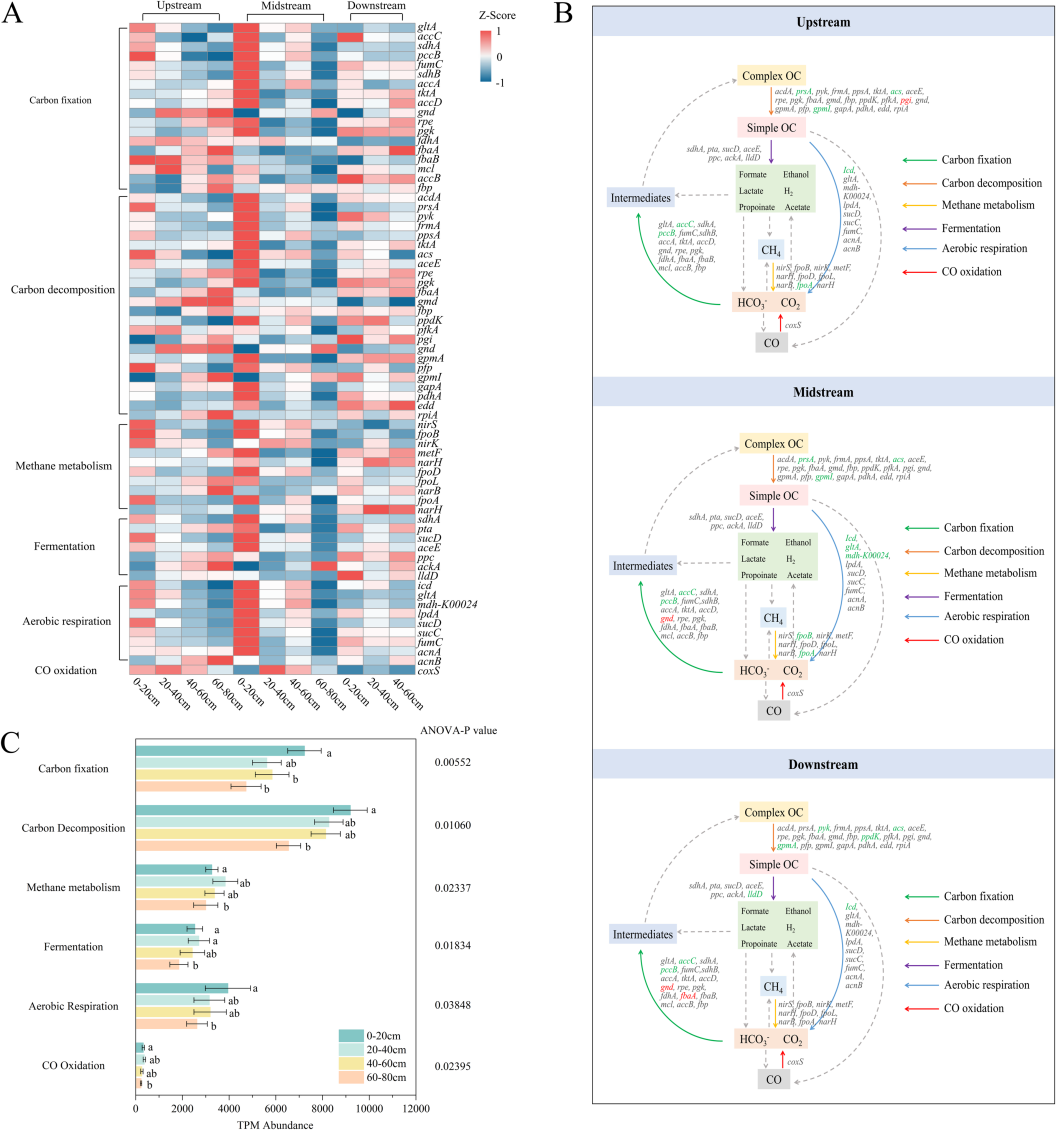
Carbon cycling abundance **(A)**, metabolic pathways **(B)**, and related gene abundance **(C)** of riparian soil microorganisms. Upstream: upstream riparian zone; Midstream: midstream riparian zone; Downstream: downstream riparian zone. 0–20 cm, 20–40 cm, 40–60 cm, and 60–80 cm represent soil samples from the corresponding depths. Arrows in different colors represent different metabolic pathways. Genes marked in green show a significant decrease with increasing depth, while genes marked in red show a significant increase with increasing depth. ANOVA was used to test the statistical differences of these metabolic pathways, with significance levels less than 0.05.

Through metagenomic analysis and annotation using the KEGG database, a total of 71 carbon metabolism functional genes (with relative abundance greater than 0.01%) were identified, primarily involving six processes: carbon fixation, carbon degradation, methane metabolism, fermentation, aerobic respiration, and carbon monoxide oxidation. These functional genes exhibited significant spatial variation in the riparian zone of semi-arid regions (Figure 3A). Overall, there was no significant trend of variation in carbon metabolism abundance between the upstream, midstream, and downstream riparian zones along the river gradient (Figure 3A; Supplementary Table S3). Carbon metabolism abundance was highest in the surface soils (0–20 cm) and lowest in the deep soils (60–80 cm) (Figures 3A,C). This suggests that microbial carbon metabolism potential is relatively balanced along the river gradient, possibly constrained by the semi-arid climate and homogeneous soil texture in the study area, or by the microbial communities becoming more homogeneous due to the influence of hydrological connectivity (Cui et al., 2019a; Yang Y. et al., 2025). In the absence of significant horizontal gradient differences, the spatial variation was primarily reflected in the vertical differences.

Carbon fixation is the primary carbon metabolic process in riparian zones of semi-arid regions. Among the genes involved in carbon fixation, *accC* and *pccB* are mainly enriched in the surface soils across all three riparian zones, and their abundance significantly decreases with increasing depth (Figure 3B; Supplementary Figure S4; Supplementary Table S3) (*p* < 0.05). This is likely closely related to the environmental characteristics of the surface soils, such as higher oxygen levels, sufficient light, and abundant organic substrate supply, which are favorable for the growth and metabolism of carbon-fixing microorganisms (Xie et al., 2021). In the wetland ecosystems of the Songnen Plain in Northeast China (Luo et al., 2025), soil carbon metabolism is dominated by organic matter decomposition and fermentation processes, with relatively weak carbon fixation gene expression, especially in high-salinity areas where carbon fixation functions are significantly inhibited. This difference may stem from the larger organic matter input and sufficient moisture in humid regions, where microorganisms tend to rely on heterotrophic decomposition (Zhou et al., 2024), while in semi-arid regions, carbon sources are limited, and environmental stress is significant. Microorganisms in semi-arid areas enhance their carbon fixation abilities to maintain energy balance and ecosystem functions (Cui et al., 2019b).

Other carbon metabolic processes also show differentiated responses to soil vertical gradients across different riparian zones. For example, in terms of carbon degradation, the abundance of *prsA*, *acs*, and *gpml* genes significantly decreases with depth in the upstream and midstream riparian zones, while genes such as *pyk*, *acs*, *ppdK*, and *gpmA* in the downstream riparian zone also show a declining trend (Figures 3A,B; Supplementary Figure S4) (*p* < 0.05). This may be due to the limited organic matter input and lower oxygen concentration in deeper soils, which restricts carbon degradation metabolism, leading to a decrease in the abundance of related functional genes (Yang Y. et al., 2025). This phenomenon is also observed in semi-arid grassland and shrubland ecosystems, where carbon metabolism gene abundance declines under low moisture and low organic carbon input conditions. In forests and wetlands with adequate moisture and high organic carbon input, decomposition metabolic activity is significantly enhanced, highlighting the high sensitivity of carbon degradation functions to moisture and organic carbon input (Fu et al., 2024; Yang R. et al., 2025).

#### 3.3.2 Vertical variation of functional genes under multiple pathways in the nitrogen cycle

**Figure 4:**
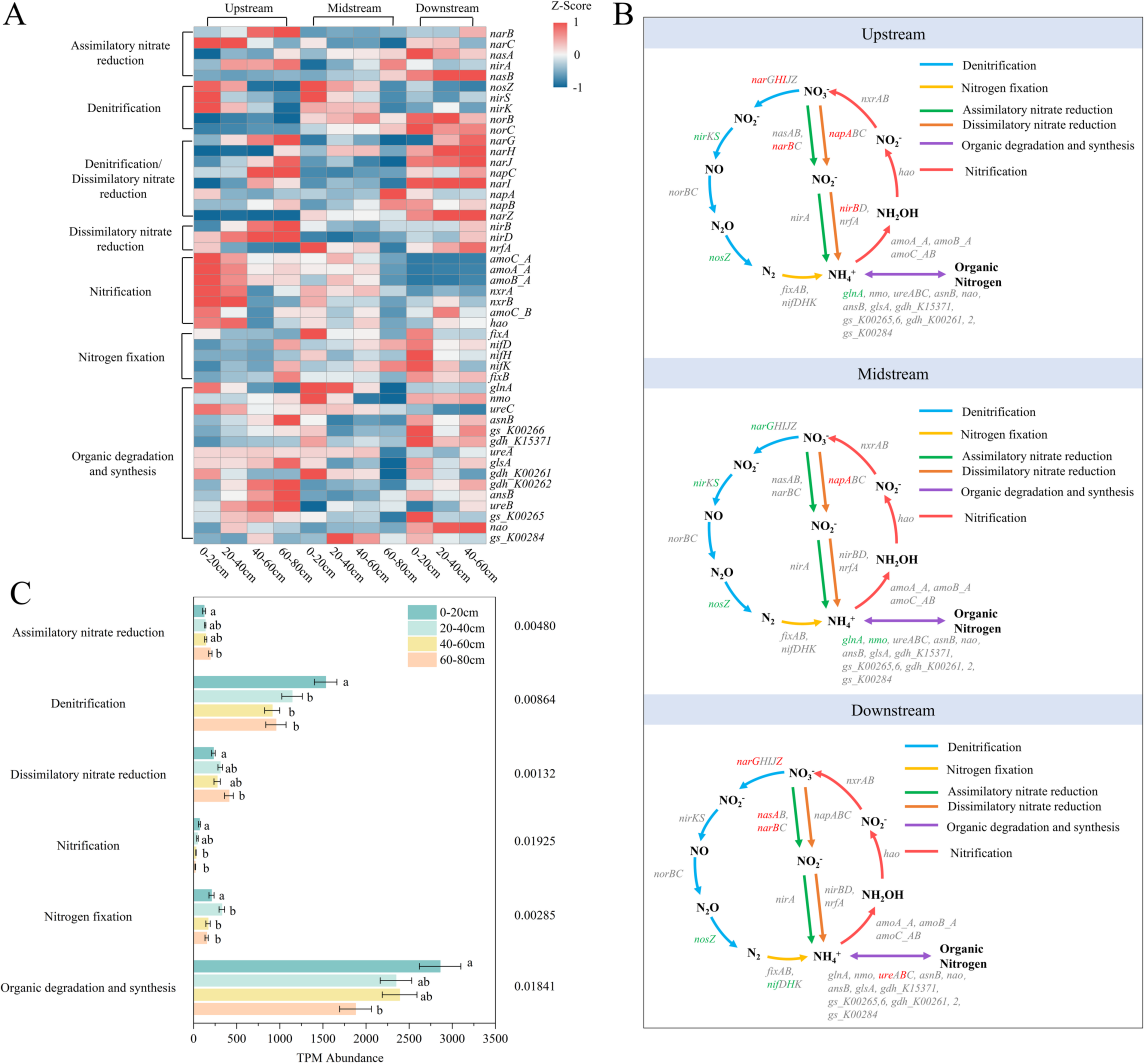
Nitrogen cycling abundance **(A)**, metabolic pathways **(B)**, and related gene abundance **(C)** of riparian soil microorganisms. Upstream: upstream riparian zone; Midstream: midstream riparian zone; Downstream: downstream riparian zone. 0–20 cm, 20–40 cm, 40–60 cm, and 60–80 cm represent soil samples from the corresponding depths. Genes marked in green show a significant decrease with increasing depth, while genes marked in red show a significant increase with increasing depth. ANOVA was used to test the statistical differences of these metabolic pathways, with significance levels less than 0.05.

Through metagenomic analysis, six nitrogen metabolism pathways were detected, including nitrogen fixation, nitrification, denitrification, organic degradation and synthesis, dissimilatory nitrate reduction, and assimilatory nitrate reduction. These functional pathways exhibited significant spatial variation in the riparian zones of semi-arid regions (Figure 4A). Overall, there was no significant trend of change in nitrogen metabolism pathways between the upstream, midstream, and downstream riparian zones along the river gradient. In the upstream riparian zone, the dominant nitrogen metabolism process was nitrification (Figures 4A,C), which may be related to its well-oxygenated environment and good surface soil permeability, conditions favorable for the enrichment of aerobic nitrifying microorganisms (Ma et al., 2025; Xiao et al., 2022). This phenomenon suggests that nitrogen metabolism pathways may be more sensitive to local environmental conditions. To further clarify the variation patterns of this response mechanism along the vertical gradient, the study focused on analyzing the vertical distribution characteristics of each nitrogen metabolism functional gene across the upstream, midstream, and downstream riparian zones (Figure 4B; Supplementary Figure S5), aiming to reveal their succession process at the site scale and their spatial heterogeneity.

Denitrification and organic nitrogen degradation and synthesis are the most dominant nitrogen metabolism processes in riparian zones of semi-arid regions. Among them, the abundance of the denitrification-related *nosZ* gene decreased with depth in all three riparian zones, while the *nirK* gene also showed a downward trend in the upstream and midstream zones (Figures 4A,B; Supplementary Figure S5; Supplementary Table S3). This phenomenon is consistent with the significantly higher ${\mathrm{NO}}_{3}^{-}-N,$ concentration in the surface soils compared to the deeper layers, providing ample electron acceptor substrates for denitrifying bacteria (Soratur et al., 2024) (*p* < 0.05). In contrast to riparian zones in semi-arid regions, in agricultural riparian zones, nitrification is the dominant process due to the abundant nutrient environment, while denitrification plays a weaker role (Lyu et al., 2023). The genes related to organic nitrogen degradation and synthesis, such as *glnA* and *nom*, showed a significant decrease with depth in the upstream and midstream riparian zones (Figures 4A,B; Supplementary Figure S5), indicating stronger nitrogen metabolism activity in the surface layers, possibly due to the accumulation of SOC and TN, as well as higher microbial diversity. In comparison, in agricultural riparian zones, the surface nitrogen metabolism genes are highly abundant in the plow layer, while in semi-arid regions, the deep metabolic potential is more controlled by environmental stress, reflecting vertical adaptive differences in the semi-arid zones (Cui et al., 2019b; Li Y. et al., 2024).

Other nitrogen metabolism processes also show differentiated responses to soil vertical gradients. For example, in the nitrogen fixation process, the *nifH* gene related to nitrogen fixation significantly decreased with depth in the downstream riparian zone, with a higher abundance in the surface soils (Figures 4A,B; Supplementary Figure S5) (*p* < 0.05). This difference may be related to the higher SOC, TN, and porosity in the surface soils, providing a favorable environment for the metabolic activity of nitrogen-fixing microorganisms (Cui et al., 2018). This differential distribution pattern is also observed in humid forest areas, where the limitation of nitrogen fixation in deeper soils is less pronounced than in the study area, indicating that nutrient scarcity in semi-arid regions has a more significant limiting effect on nitrogen fixation capacity (Levy-Booth et al., 2014).

#### 3.3.3 Depth dependent distribution of phosphorus related genes dominated by purine and pyrimidine metabolism

**Figure 5:**
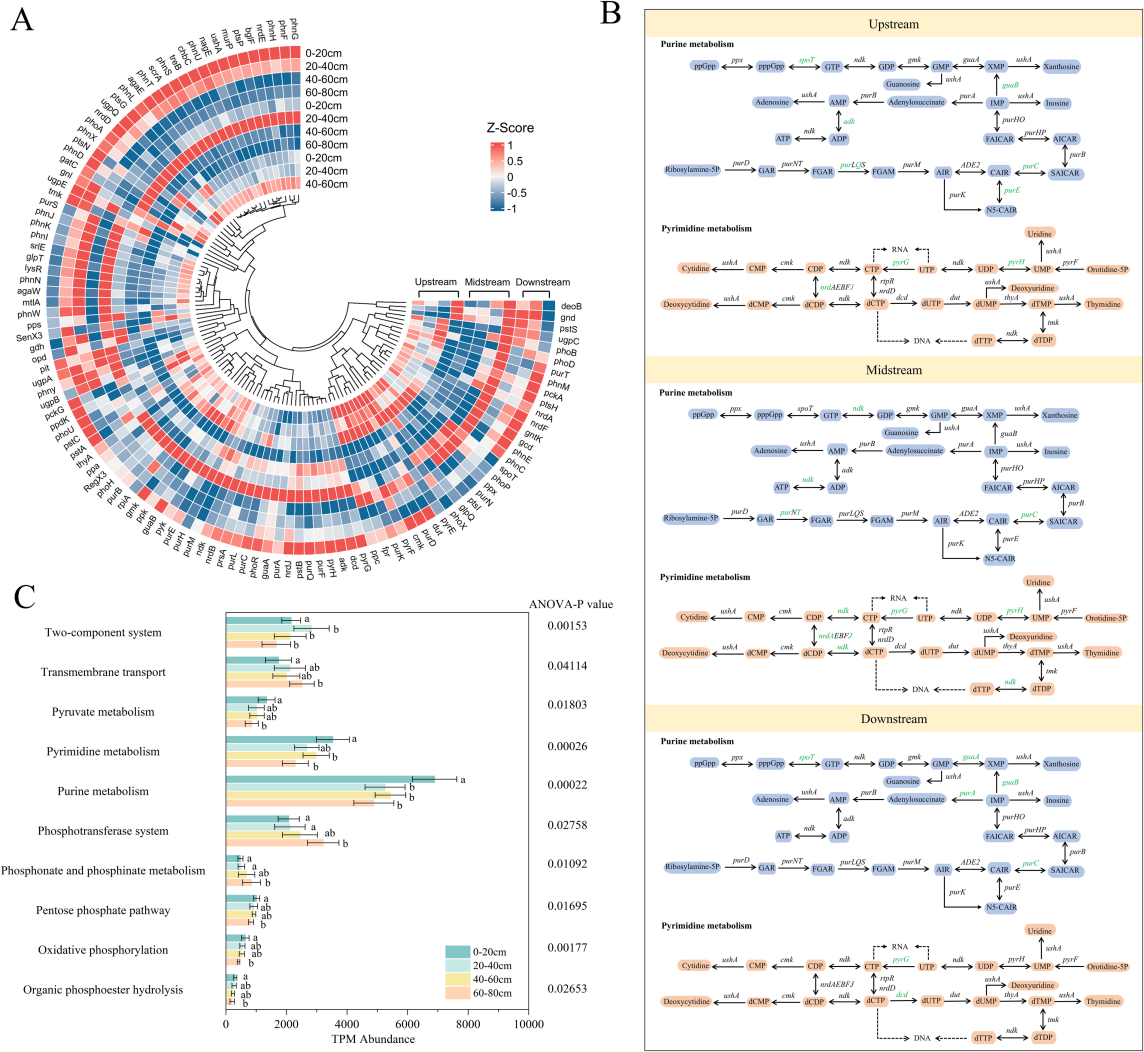
Phosphorus cycling abundance **(A)**, metabolic pathways **(B)**, and related gene abundance **(C)** of riparian soil microorganisms. Upstream: upstream riparian zone; Midstream: midstream riparian zone; Downstream: downstream riparian zone. 0–20 cm, 20–40 cm, 40–60 cm, and 60–80 cm represent soil samples from the corresponding depths. Arrows in different colors represent different metabolic pathways. Genes marked in green show a significant decrease with increasing depth, while genes marked in red show a significant increase with increasing depth. ANOVA was used to test the statistical differences of these metabolic pathways, with significance levels averaging less than 0.05.

Through metagenomic functional annotation, a total of 10 phosphorus metabolism pathways were identified, including purine metabolism, pyrimidine metabolism, two-component systems, phosphotransferase systems, transmembrane transport, pyruvate metabolism, pentose phosphate pathway, phosphonate and phosphinate metabolism, oxidative phosphorylation, and organic phosphate ester hydrolysis. A total of 112 phosphorus metabolism-related functional genes were detected across these pathways (Figure 5A). Overall, there was no significant change in phosphorus metabolism functional genes along the river gradient, but a systematic gradient variation was observed vertically (Figures 5B,C; Supplementary Table S4), indicating that vertical environmental differences are the main factors driving the changes in phosphorus metabolism potential in this region. This contrasts with findings from riparian zones in humid regions, where phosphorus metabolism typically shows gradient changes along the river due to hydrological connectivity and vegetation input. In this study area, the semi-arid climate and homogeneous soil texture conditions weakened the differences along the river gradient (Geng et al., 2022; Wang L. L. et al., 2022).

Purine metabolism and pyrimidine metabolism are the dominant phosphorus metabolic processes in riparian zones of semi-arid regions. Among them, key purine metabolism genes such as *spoT* and *guaB* significantly decreased with depth in the upstream and downstream riparian zones, and *purC* showed a significant decreasing trend across all three river sections (Figures 5B,C; Supplementary Figure S6; Supplementary Table S4) (*p* < 0.05), indicating that purine metabolism activity is strongest in the surface soils. The pyrimidine metabolism-related gene *pyrG* decreased significantly from the surface to the deeper layers in all river sections, while *pyrH* and *nrdJ* showed significant declines in the upstream and midstream zones, and dcd exhibited the most noticeable change in the downstream zone (Figures 5A,B; Supplementary Figure S6) (*p* < 0.05). This differs from humid regions, where the dominant phosphorus metabolism processes are often organic phosphorus mineralization and inorganic phosphorus dissolution, and these processes can maintain relatively high activity in deeper soils, which may be linked to the higher organic matter input in humid regions (Liu Z. et al., 2024). In semi-arid regions, phosphorus metabolism functions significantly decline in deeper soils due to water and nutrient scarcity. This difference reflects the adaptive variations in phosphorus metabolism pathways between humid and semi-arid regions, where the humid regions, with abundant water resources, can support more water-dependent phosphorus metabolism processes, while semi-arid regions rely primarily on adaptive mechanisms for water conservation and low phosphorus availability (Chen et al., 2024; Li Y. et al., 2024).

Other phosphorus metabolic processes also show differentiated responses to soil vertical gradients. In the resource-limited deeper soils, the abundance of phosphotransferase system (PTS) and transmembrane transport-related functional genes significantly increased (Figure 5A; Supplementary Figure S6) (*p* < 0.05), suggesting that microorganisms enhance their ability to absorb and transport both inorganic and organic phosphorus to cope with phosphorus scarcity (Siles et al., 2022). In the phosphonate and phosphinate metabolism pathways, the high abundance of related genes reflects an increased potential of microorganisms to utilize less degradable phosphorus sources (Figure 5A; Supplementary Figure S6). This metabolic adaptation is highly consistent with the functional strategies of deep-layer microorganisms in no-till farmland under low phosphorus availability conditions, reflecting the resilience mechanisms of microorganisms in semi-arid regions (Bai et al., 2025; Wang X. et al., 2024).

### 3.4 Environmental drivers of microbial community structure and metabolic functions

To further reveal the environmental driving mechanisms of microbial community structure and their carbon, nitrogen, and phosphorus metabolic functions in riparian zones of semi-arid regions, the study used the Partial Mantel test to systematically analyze the correlations between microbial community structure, functional metabolism, and soil physicochemical factors. The study established a multi-layered statistical analysis framework centered on the Partial Mantel test. This method was employed to assess the correlations between ecological dissimilarity matrices while controlling for potential confounding variables. Bray–Curtis dissimilarity was used to quantify the variation in microbial community composition and functional gene profiles, whereas Euclidean distance was applied to represent differences in environmental variables. To reduce multicollinearity and enhance the robustness of the analysis, variance inflation factor (VIF) screening was incorporated, and only variables with VIF values less than 10 were retained for subsequent modeling.

**Figure 6:**
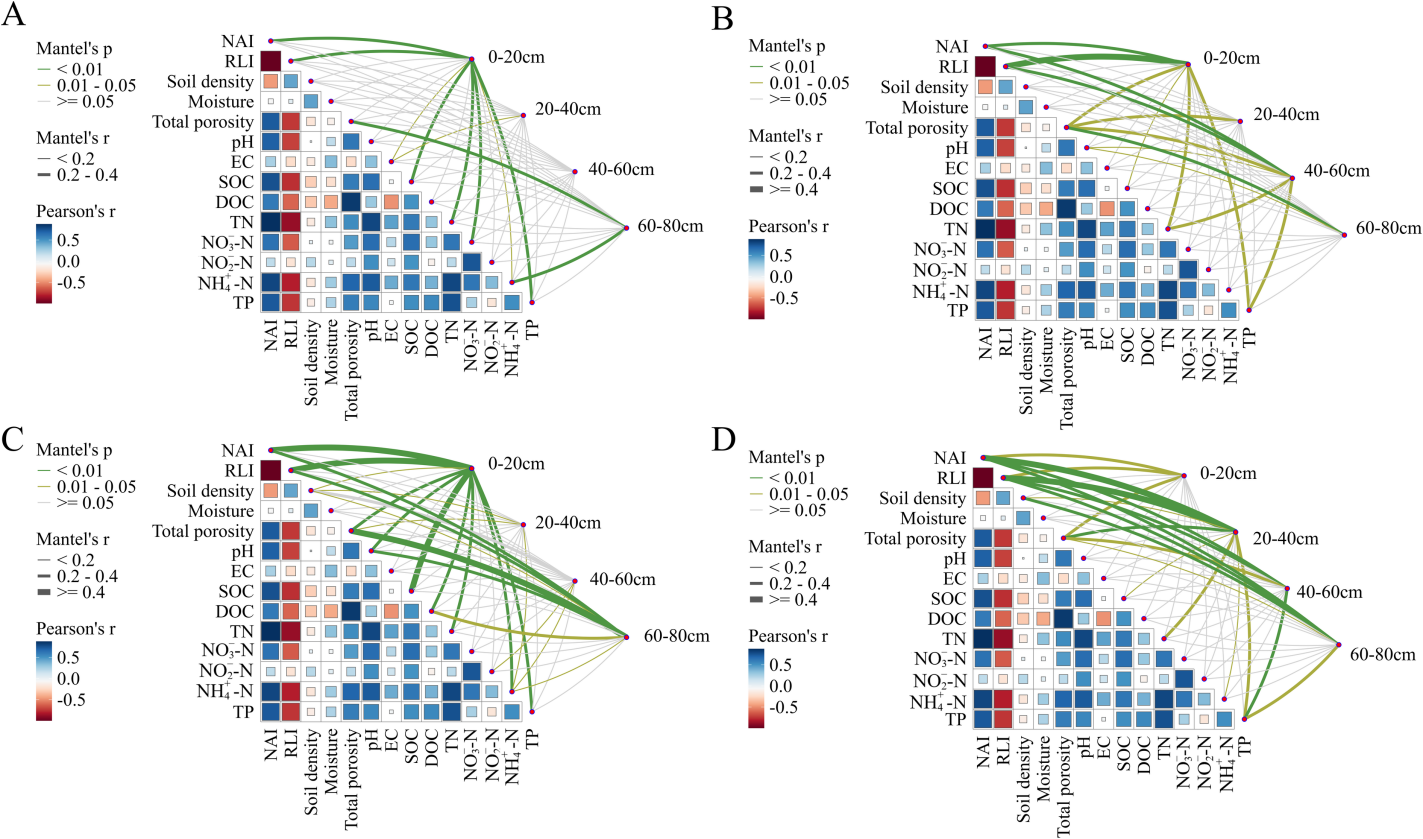
Correlation between microbial community structure **(A)** (determined by bray-curtis distance), carbon **(B)**, nitrogen **(C)**, and phosphorus **(D)** metabolism (determined by functional genes) with environmental factors, using the partial Mantel test. The partial Mantel’s r values are represented by edge width, and statistical significance is indicated by edge color. Pairwise correlations of environmental variables are shown using a color gradient reflecting the Spearman correlation coefficient. 0–20 cm, 20–40 cm, 40–60 cm, and 60–80 cm represent soil samples from the corresponding depths.

The analysis results showed that in the upstream, midstream, and downstream riparian zones, microbial community structure and its carbon, nitrogen, and phosphorus metabolic functions were significantly correlated with soil depth, porosity, and nutrient factors such as SOC, TN, and TP (Supplementary Figure S7, *p* < 0.05, r > 0.2), confirming that microbial ecological processes are significantly regulated by soil physicochemical characteristics at the watershed scale. Furthermore, the results indicated that within different soil depth intervals, the environmental factors dominating microbial structure and functional expression exhibited distinct differences (Figure 6).

From the perspective of microbial community structure, the microbial community in surface soils (0–20 cm) showed a significant positive correlation with NAI, RLI, SOC, TN, ${\mathrm{NO}}_{3}^{-}-N,$ and TP (Figure 6A, *p* < 0.05, r > 0.2). These nutrients provide carbon and nitrogen sources for microorganisms, effectively supporting their proliferation and maintaining diversity, especially since SOC and TN are abundant in the surface soils (Figure 1), making them core factors in regulating microbial metabolic activity (Banerjee et al., 2018). In deep soils (60–80 cm), the microbial community structure showed a stronger correlation with porosity, reflecting that, under conditions of limited nutrient input, soil structural characteristics may become an important limiting factor influencing microbial distribution and physiological activities (Yang C. et al., 2019).

Microbial carbon metabolism functions exhibit a hierarchical response to soil environments. In the surface soils, carbon metabolism functions were significantly correlated with NAI, RLI, TN and TP, indicating that nitrogen and phosphorus supply plays a synergistic role in regulating carbon metabolism (Yu et al., 2025). In contrast, in the deeper soils (40–80 cm), carbon metabolism functions were more dependent on factors such as porosity, DOC, and pH, with porosity having the most significant influence (Figure 6B, *p* < 0.05, r > 0.2). This suggests that under nutrient-poor, low-oxygen conditions, microbial metabolic activity becomes more dependent on soil aeration and the availability of organic carbon (Hill et al., 2019; Yang J. et al., 2019).

The response of nitrogen metabolism functions to the soil environment also shows clear vertical differentiation. In the surface soils (0–20 cm), nitrogen metabolism was closely correlated with porosity, NAI, RLI, pH, SOC, DOC, TN, and ${\mathrm{NH}}_{4}^{+}-N$ (Figure 6C, *p* < 0.05, r > 0.2), reflecting the microorganisms’ integrated response to organic matter, nitrogen sources, and pH conditions (Ye et al., 2024). In the 20–40 cm layer, nitrogen metabolism was significantly influenced by bulk density, moisture content, and electrical conductivity, indicating that the physicochemical structure of this layer regulates microbial nitrogen transformation pathways (Yang C. et al., 2025). In deeper soils (60–80 cm), nitrogen metabolism was most strongly correlated with porosity, pH, and ${\mathrm{NH}}_{4}^{+}-N,$ suggesting that under low-oxygen and resource-limited conditions, microorganisms tend to maintain nitrogen cycling through anaerobic nitrogen reduction pathways (Wang and Kuzyakov, 2024).

Phosphorus metabolism functions are regulated by a combination of factors. In the surface soils, phosphorus metabolism was closely related to bulk density and porosity (Figure 6D, *p* < 0.05, r > 0.2), with low bulk density and good porosity structure facilitating oxygen diffusion and microbial activity, thereby promoting aerobic phosphorus metabolism (Gul et al., 2015). With increasing depth, NAI, RLI, TP and TN became the key influencing factors, especially in the 40–80 cm layers, indicating that in environments with limited carbon sources, microorganisms rely on the coupling of endogenous phosphorus and nitrogen to regulate their phosphorus metabolism functions (Cui et al., 2022; Wang H. et al., 2024).

Notably, among all observed relationships between microbial functions (C, N, and P metabolism) and environmental factors, nutrient availability and porosity consistently emerged as the key influencing variables. Specifically, the Nutrient Availability Index (NAI) played a dominant role in surface soils, while porosity served as a critical regulatory factor in deeper layers. This suggests that soil structural properties not only regulate gas diffusion and water movement but also have a profound impact on microbial niche structure, energy metabolism, and functional gene expression (Li Z. et al., 2024). Meanwhile, nutrient indicators such as SOC, TN, and TP primarily affect surface soils, with their influence decreasing with depth. This reflects that nutrient availability plays a dominant role in shallow soil microbial metabolism, while deeper soils rely more on the support of soil physical environments (Naylor et al., 2022; Spohn et al., 2016). This highlights the joint role of nutrient availability and soil structure in driving the spatial distribution of microbial metabolic functions.

### 3.5 Microbial response patterns from the ecological function perspective

In the riparian ecosystems of the semi-arid regions, microorganisms, as key driving factors, reflect the effectiveness of ecological interventions and environmental adjustments through changes in their structure and functions (Shu and Huang, 2022). In this study, ecological functions refer to specific microbial activities that regulate nutrient cycling, such as carbon fixation, nitrification, denitrification, and purine and pyrimidine metabolism. These functions are essential for maintaining the stability of riparian ecosystems and supporting biogeochemical cycles. We represent these ecological functions through the abundance of key functional genes related to carbon, nitrogen, and phosphorus cycling. Gene abundance was quantified using metagenomic sequencing and normalized by transcripts per million (TPM) to account for sequencing depth and gene length.

At the watershed scale, microbial responses to ecological functions show spatial selectivity. Studies have shown that between the upstream, midstream, and downstream riparian zones of the Tuwei River, despite similar overall environmental conditions, certain microbial groups still exhibit differentiated distribution patterns. Notably, genera such as *Sphingopyxis* and *Hydrogenophaga* show an enrichment trend in the midstream and downstream zones (Figure 2C), indicating that these areas have greater microbial rebuilding potential during the ecological restoration process (Li W. et al., 2024). The spatial heterogeneity of microbial communities may reflect the redistribution of microbial niches at the watershed scale, demonstrating the adaptive reorganization of microorganisms in response to restoration interventions (Guseva et al., 2022). Furthermore, the enrichment of key functional genes involved in nitrogen cycling, such as *nosZ* and *nirK* for denitrification, *accC* and *pccB* for carbon fixation in the carbon cycle, and *purC*, *guaB*, *pyrG* for purine and pyrimidine metabolism in the phosphorus cycle, in the downstream region (Figures 3–5) also suggests that the restoration process may involve the optimization and reorganization of microbial metabolic functions, contributing to the enhancement of overall ecosystem self-regulation and material cycling capacity (Deng et al., 2021; Wang M. et al., 2022).

At the site scale, microbial communities and their metabolic functions exhibit a more gradient-based and indicative response to ecological restoration. In the early stages of ecological restoration, the physical and chemical properties of the surface soils, such as organic matter and nutrient levels, are typically prioritized for improvement. This change is reflected at the microbial level by an increase in community diversity and enhanced functional gene activity (Xin et al., 2023). The surface soil, as the main site of microbial activity, shows intense metabolic network operation under the restoration context, becoming crucial for maintaining the carbon, nitrogen, and phosphorus cycles and supporting plant reconstruction (Spohn et al., 2016). While deep soils are less affected by intervention in the early stages of restoration, leading to simplified microbial communities, reduced metabolic capacity, and limited expression of functional microorganisms. However, as surface soil restoration progresses and soil air-water permeability improves, deep soil environments gradually become more suitable. Microbial populations such as *Hydrogenophaga* and *Bradyrhizobium*, known for their resilience, may begin to expand their metabolic activity range (Figures 2B,C), showing signs of functional activation driven by the restoration process (Li W. et al., 2022; Zhao et al., 2019). Microbial responses to ecological restoration are not synchronized across different soil layers, but rather occur progressively, extending ecological functions vertically.

Soil ecological functions are not solely dependent on microbial community structure, but are also constrained by the compatibility of environmental conditions. In surface soils, the increase in nutrient levels such as SOC and TN significantly enhances microbial metabolic activity, while in deeper soils, improvements in porosity, pH, and nutrient conditions are key to driving the expansion of microbial functions (Liu Y. et al., 2024; Philippot et al., 2024). In actual ecological restoration processes, it is important to focus on optimizing soil structural characteristics and improving the accessibility of deep soil resources, gradually building a microbial functional continuum from the surface to the deeper layers. This multi-layered response mechanism highlights the sensitive and indicative role of microbial communities in ecological restoration and provides an important biological criterion for evaluating the effectiveness of ecological restoration in riparian zones of semi-arid regions. In future ecological restoration efforts, differentiated management measures can be developed based on watershed location and soil layer characteristics to accurately activate microbial ecological functions, achieving scientifically controlled restoration processes.

## 4 Conclusion

This study elucidates the vertical differentiation and environmental drivers of microbial community structure and their associated carbon, nitrogen, and phosphorus metabolic functions in semi-arid riparian soils. We demonstrate that microbial diversity and functional gene abundance markedly decline with increasing depth, with surface soils (0–20 cm) harboring richer communities and elevated expression of key functional genes, including *accC*, *nosZ*, *nirK*, *purC*, *guaB*, and *pyrG*. Results from the Partial Mantel test indicate that nutrient availability and porosity are the principal factors shaping these spatial patterns, with porosity exerting a dominant influence in deeper layers. Importantly, the results provide actionable insights for ecological restoration. The observed depth-dependent microbial stratification suggests that improving surface soil structure and increasing nutrient accessibility may effectively stimulate microbial driven biogeochemical processes. In watershed management, microbial indices derived from key taxa or functional genes can serve as sensitive indicators for assessing ecological health, particularly in arid environments where abiotic stress is prevalent. Moreover, our findings contribute to climate change adaptation strategies by identifying microbial functions, especially carbon fixation and denitrification, that are vulnerable to environmental degradation. Future studies should combine multi-omics techniques and hydrological modeling to monitor the dynamic responses of microbial metabolic functions under seasonal and human disturbances, thereby advancing process-based restoration and adaptive management strategies in semi-arid riparian ecosystems.

## Data Availability

The data presented in this study are publicly available in figshare under the following DOI: 10.6084/m9.figshare.30796643.

## Footnotes

1
http://www.usadellab.org/cms/uploads/supplementary/Trimmomatic

2
http://www.ncbi.nlm.nih.gov/COG/

3
http://www.genome.jp/kegg/

4
https://ccycdb.github.io/

5
https://github.com/qichao1984/NCyc/tree/master/data

6
https://github.com/ZengJiaxiong/Phosphorus-cycling-database

7
https://www.genome.jp/tools/kofamkoala/

8
https://www.ebi.ac.uk/Tools/hmmer/search/hmmsearch
