# Supplementary material for: Depth differentiation of microbial communities and nutrient cycling functional genes in semi-arid riparian soil
Source: Front Microbiol. 2025 Dec 18;16:1717707. doi: 10.3389/fmicb.2025.1717707 (PMC12756892; doi:10.3389/fmicb.2025.1717707)
Supplement: Supplementary file 1 [file Data_Sheet_1.DOCX]

***Supplementary Material***

1. **Supplementary Figures and Tables**

## Supplementary Figures


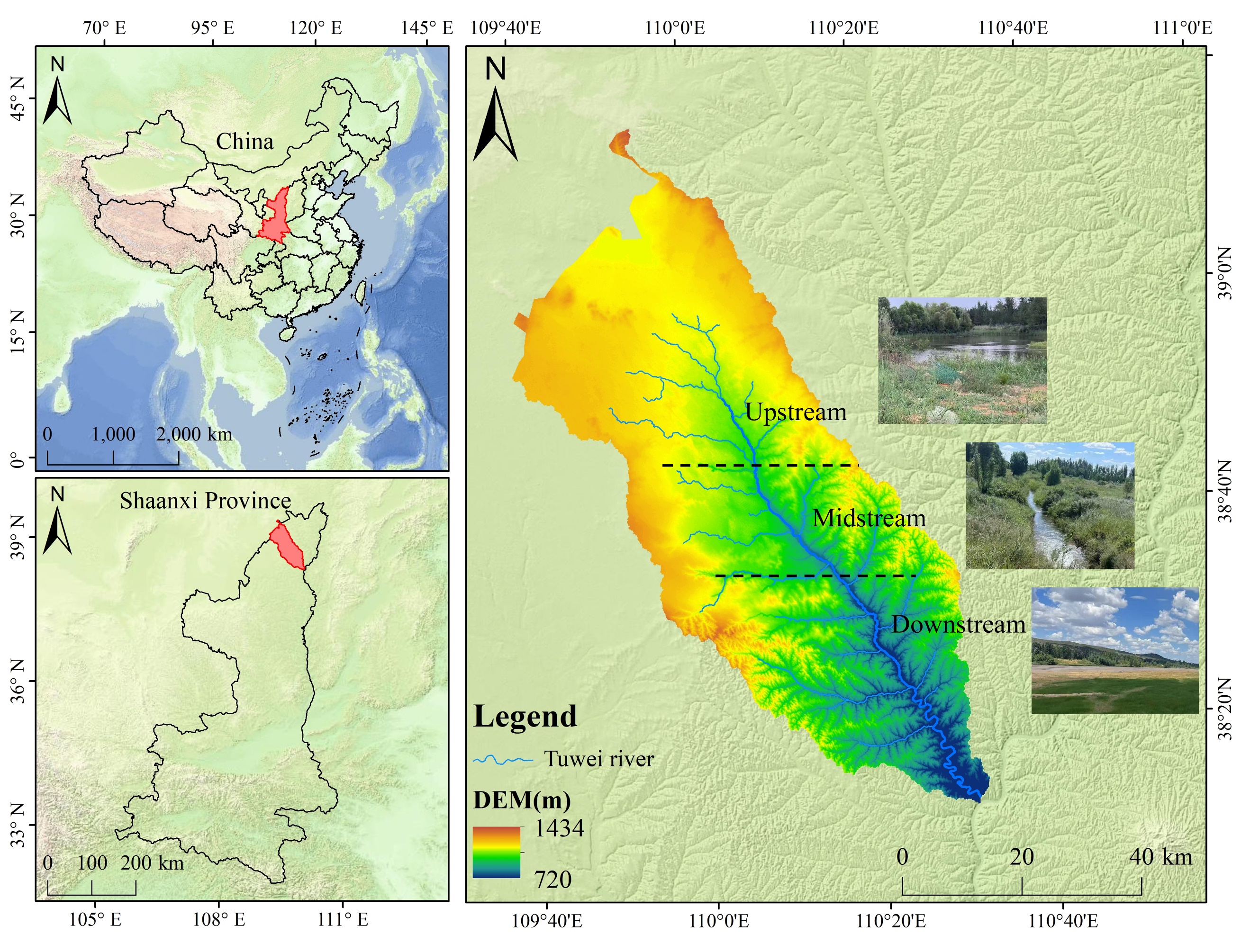


**Figure S1**: Tuwei River study area. From top to bottom are the upstream, midstream, and downstream sections.


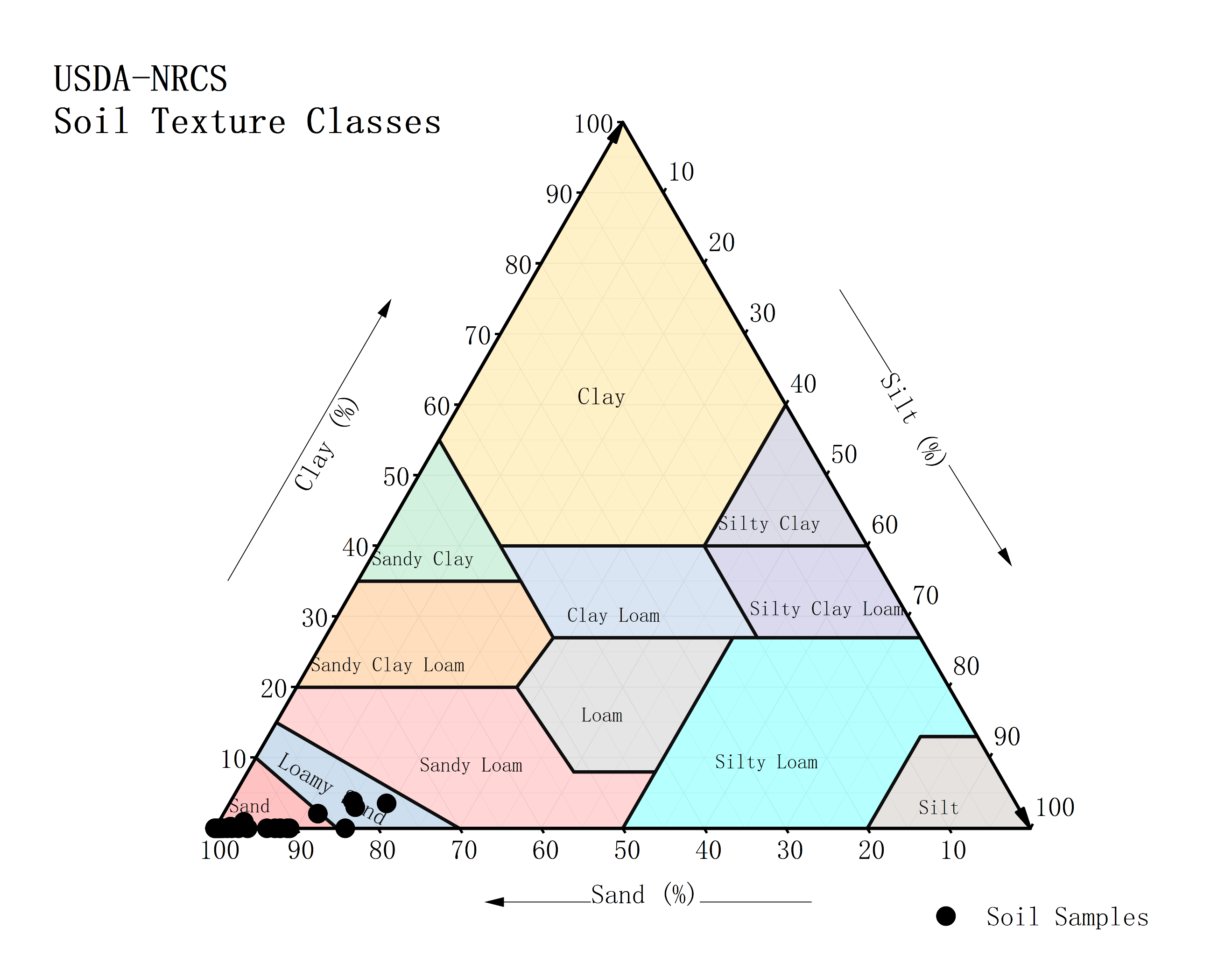


Figure S2. Soil texture classification of riparian zone soils based on sand, silt, and clay content. Each point represents a soil sample.

**
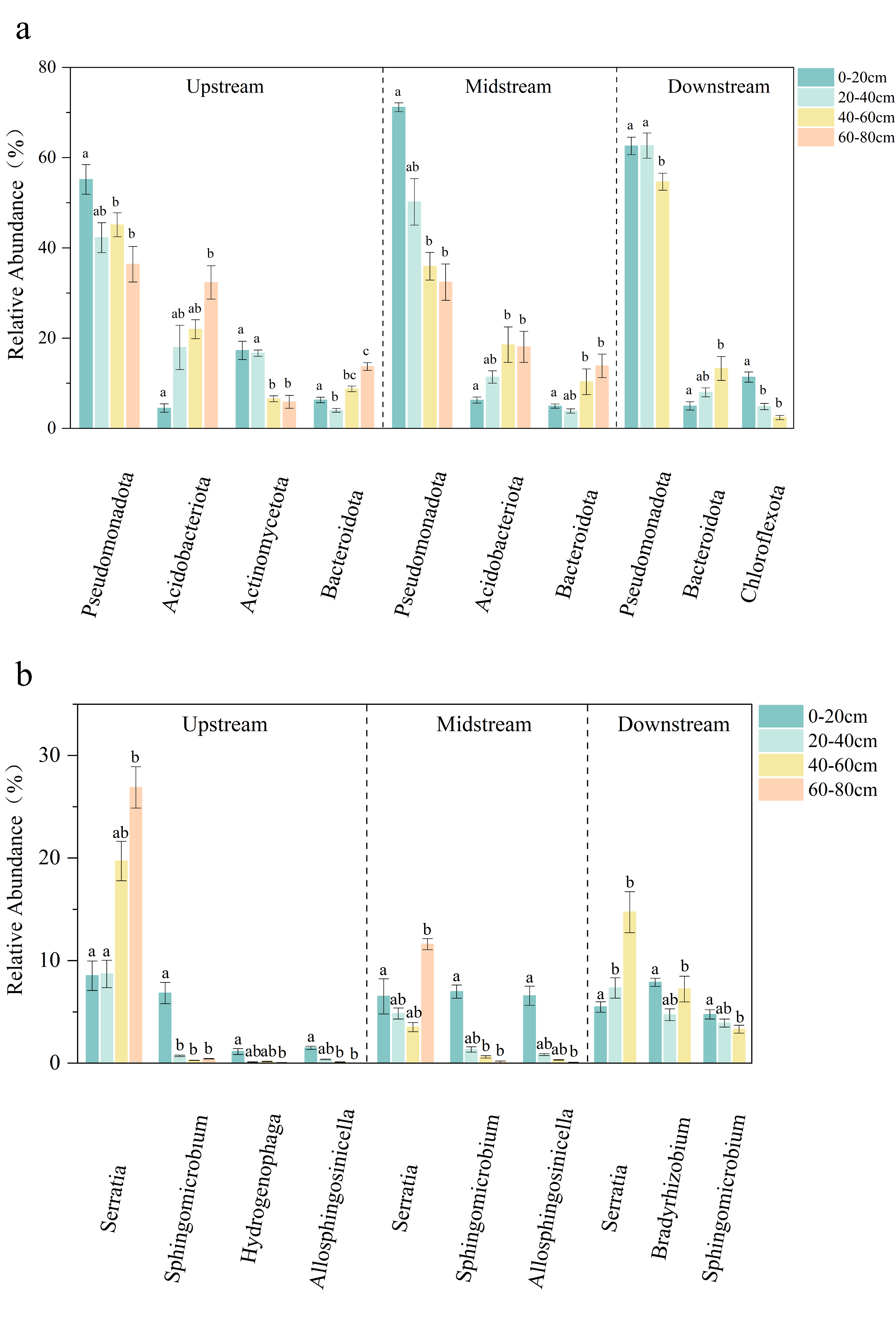
**

**Figure S3.** Relative abundance of soil microbial phylum (a) and genus (b) at different depths in the upstream, midstream, and downstream riparian zones. Upstream: upstream riparian zone; Midstream: midstream riparian zone; Downstream: downstream riparian zone. 0-20 cm, 20-40 cm, 40-60 cm, and 60-80 cm represent soil samples from the corresponding depths. Different letters indicate significant differences, p < 0.05.

**
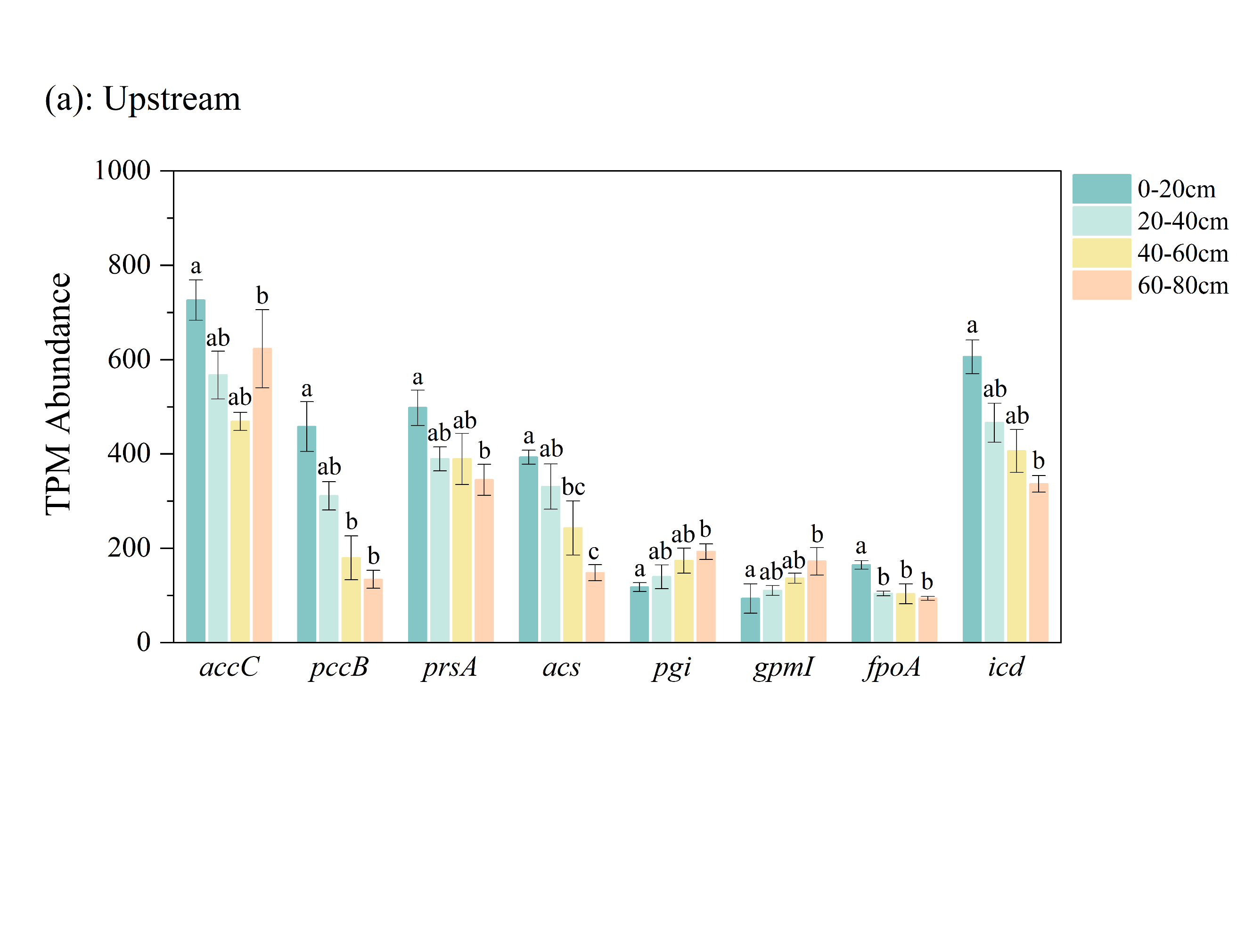
**

**
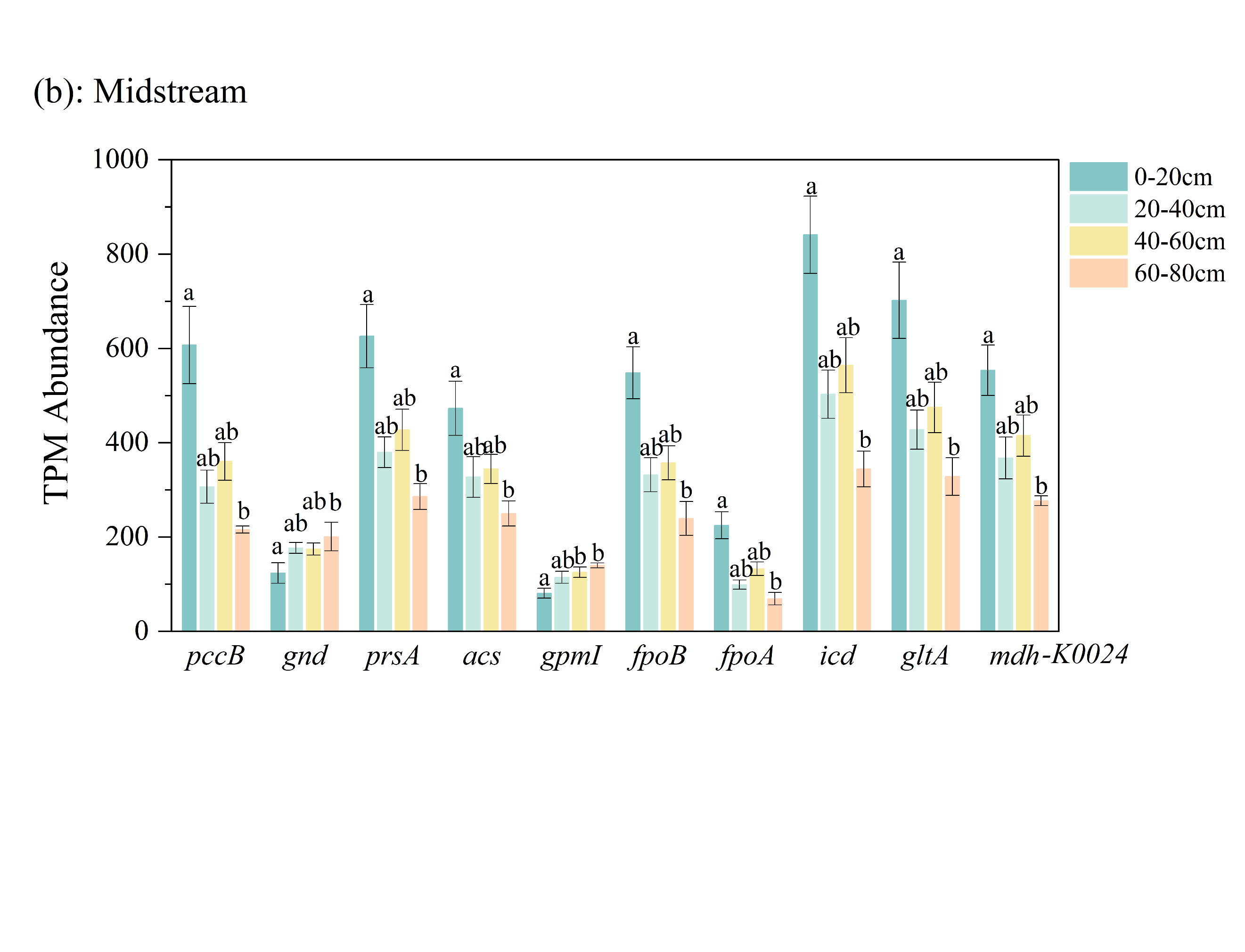
**

**
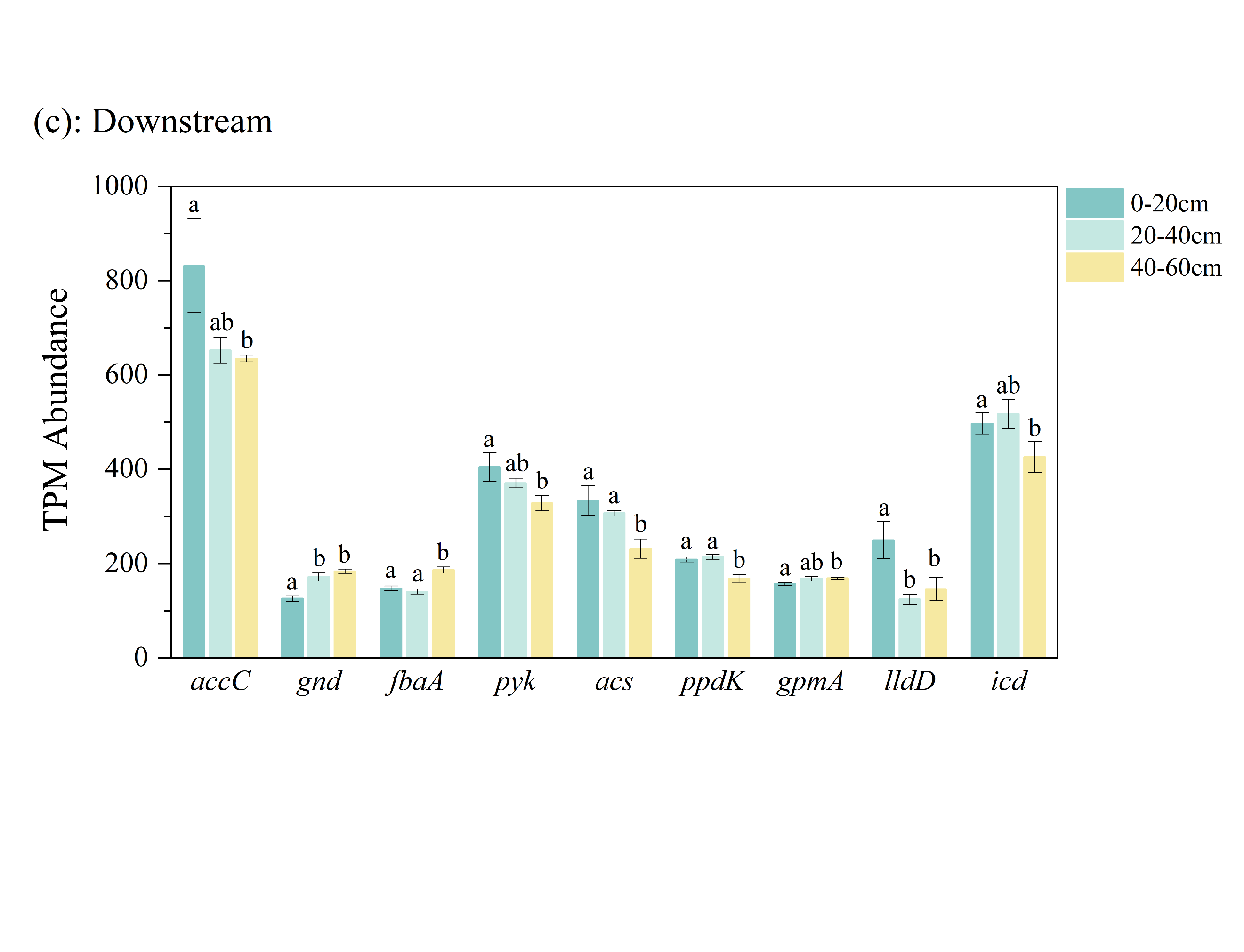
**

**Figure S4.** Abundance of functional genes of carbon metabolism at different depths in the upstream (a), midstream (b), and downstream (c) riparian zones. Only carbon metabolism genes showing significant differences across sampling layers (p < 0.05) were included in the bar chart. Upstream: upstream riparian zone; Midstream: midstream riparian zone; Downstream: downstream riparian zone. 0-20 cm, 20-40 cm, 40-60 cm, and 60-80 cm represent soil samples from the corresponding depths. Different letters indicate significant differences, p < 0.05.

**
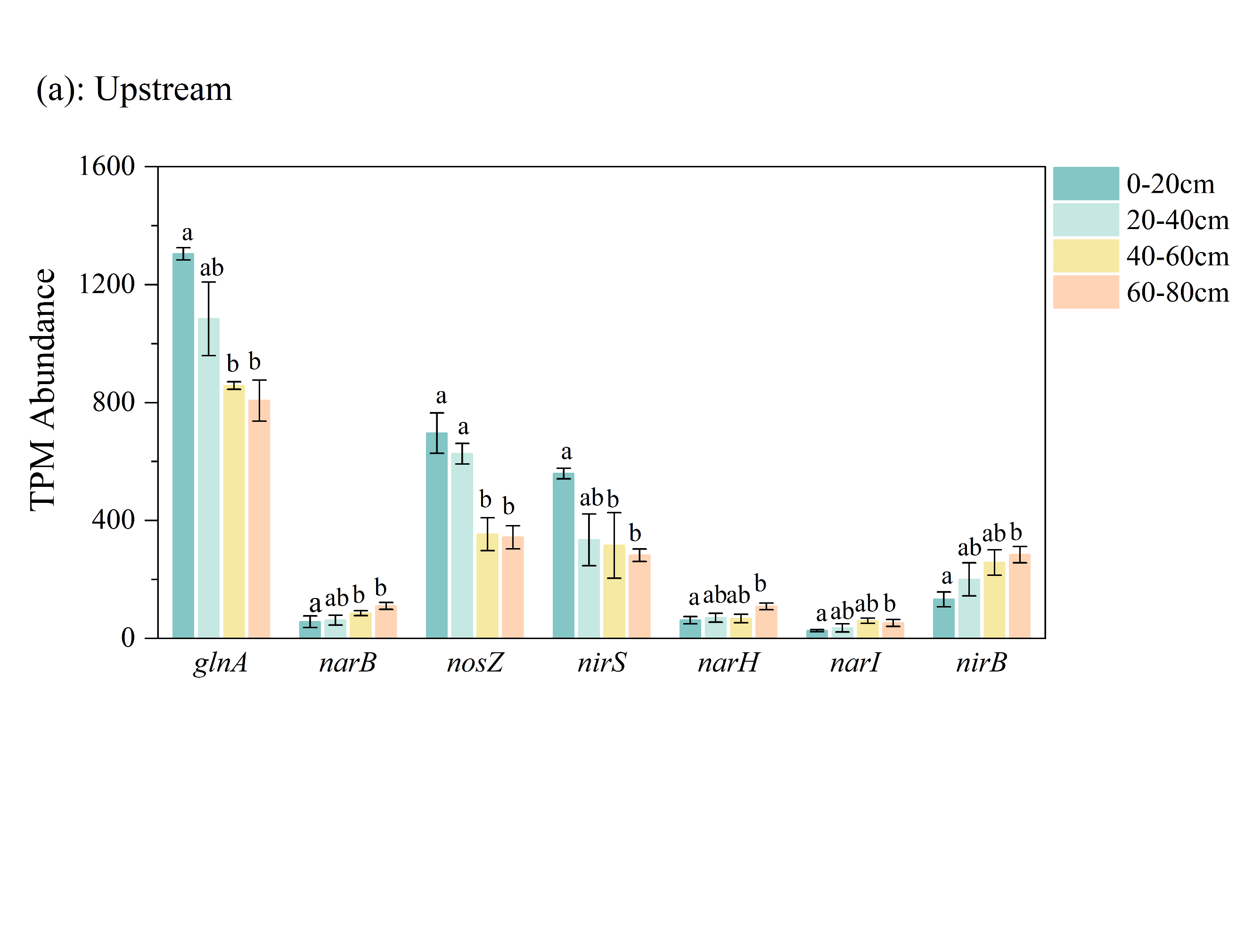
**

**
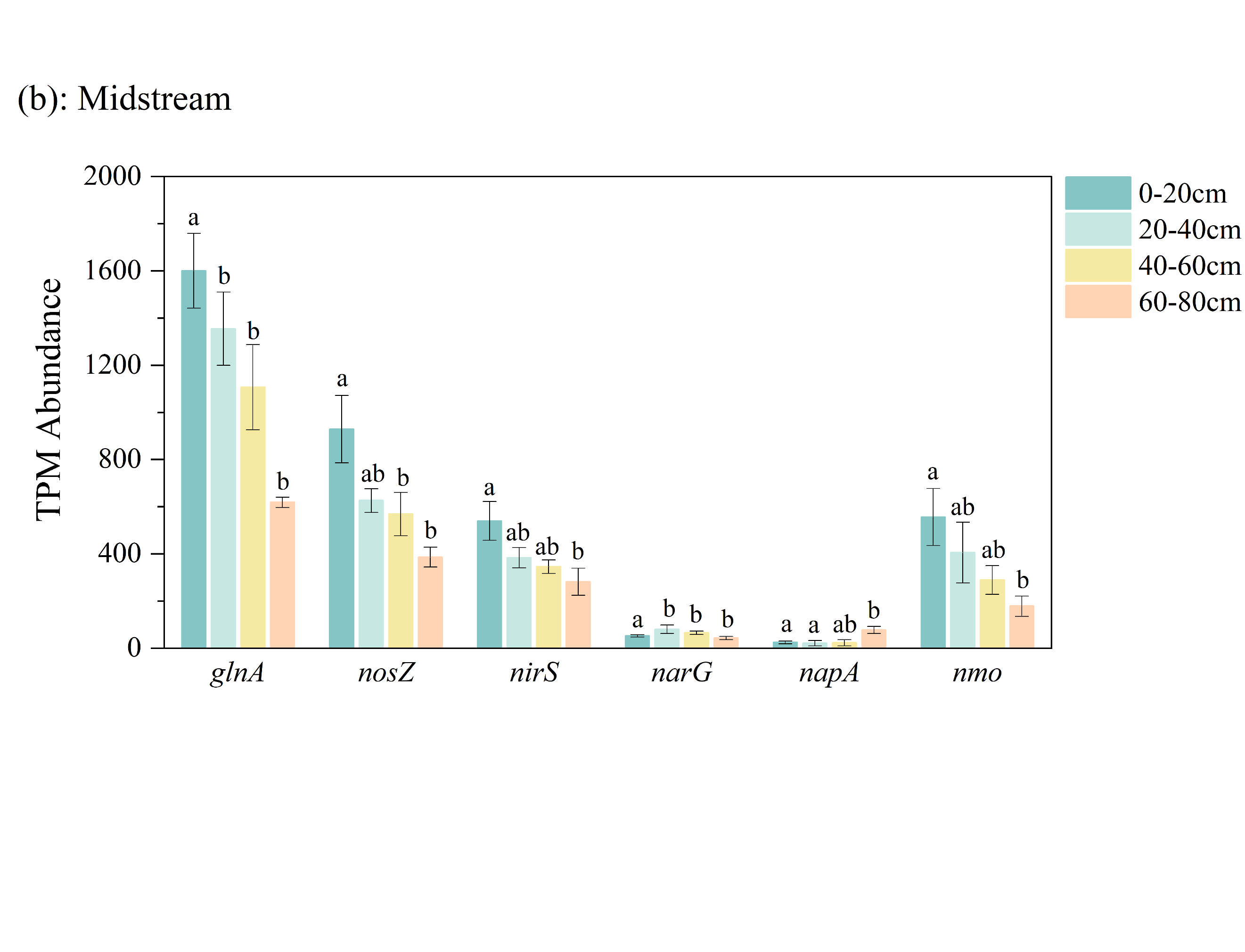
**

**
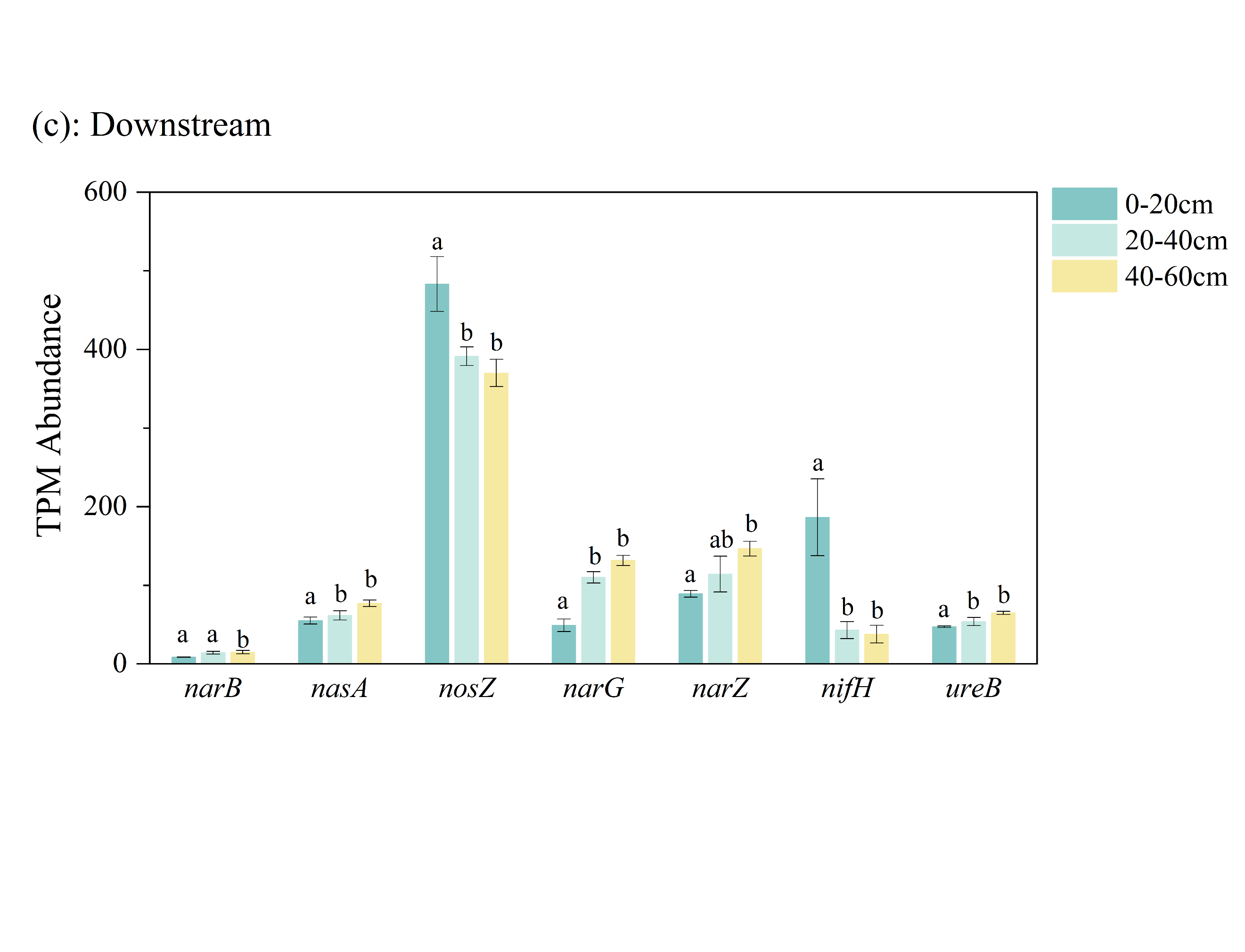
**

**Figure S5.** Abundance of functional genes of nitrogen metabolism at different depths in the upstream (a), midstream (b), and downstream (c) riparian zones. Upstream: upstream riparian zone; Midstream: midstream riparian zone; Downstream: downstream riparian zone. 0-20 cm, 20-40 cm, 40-60 cm, and 60-80 cm represent soil samples from the corresponding depths. Different letters indicate significant differences, p < 0.05.

**
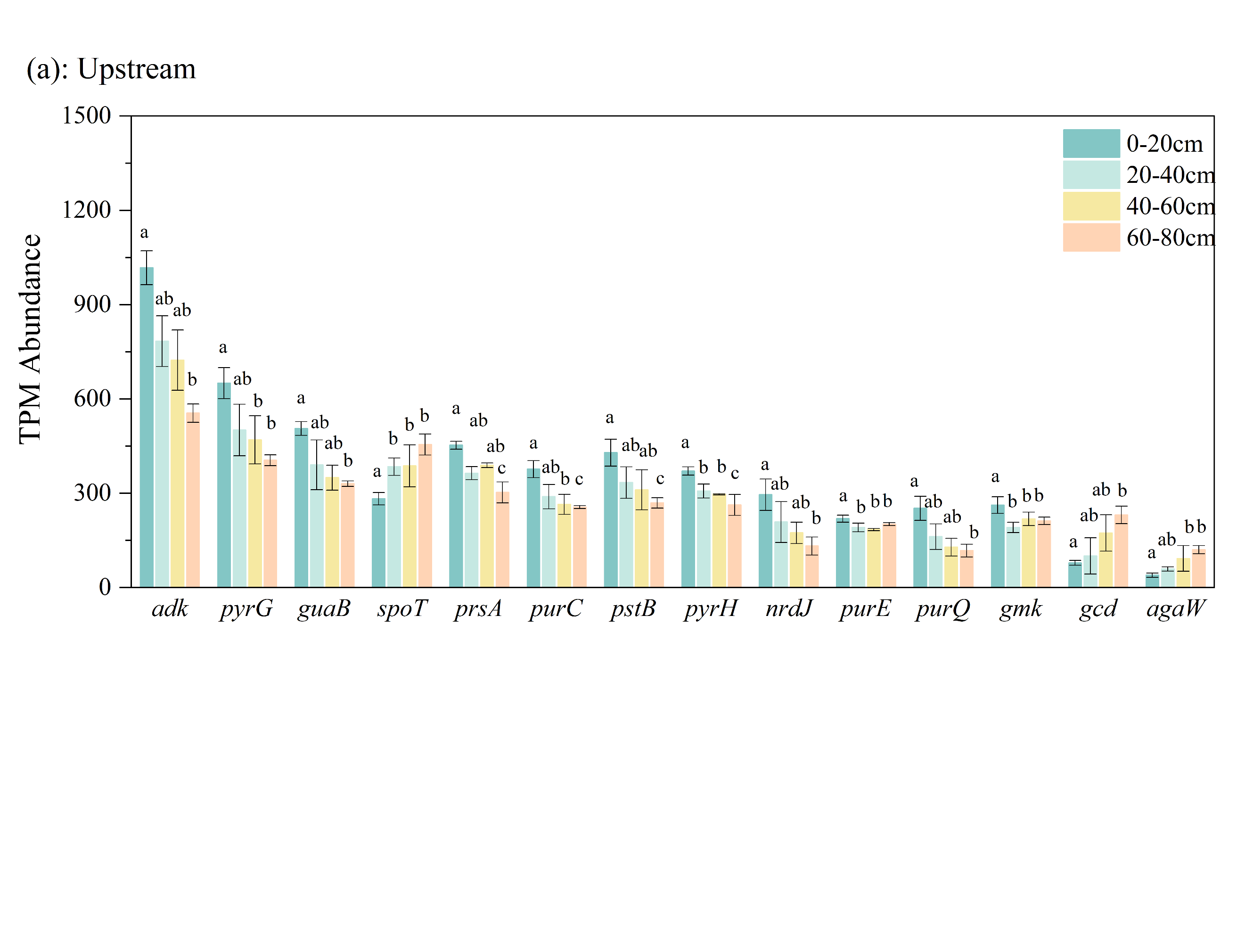
**

**
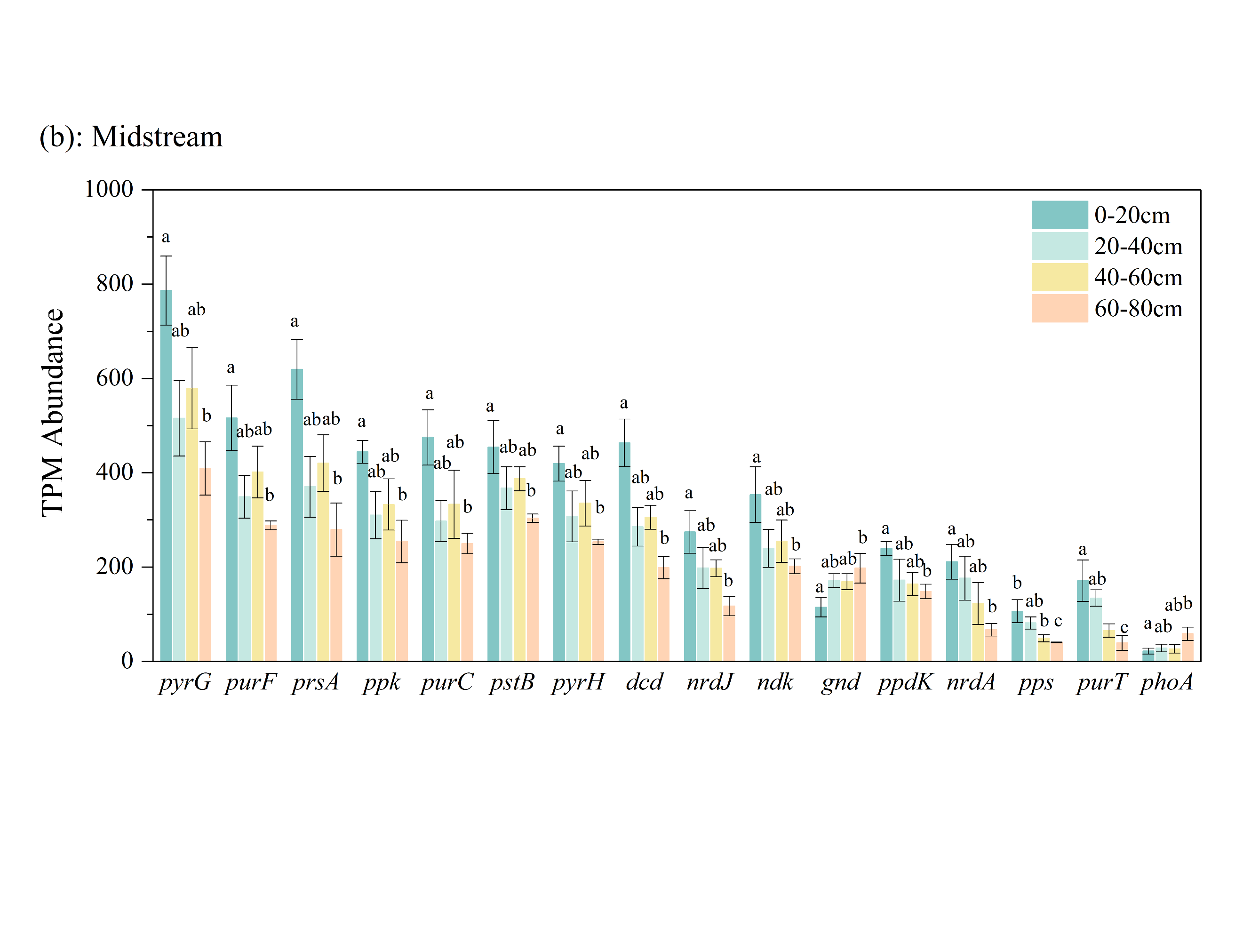
**

**
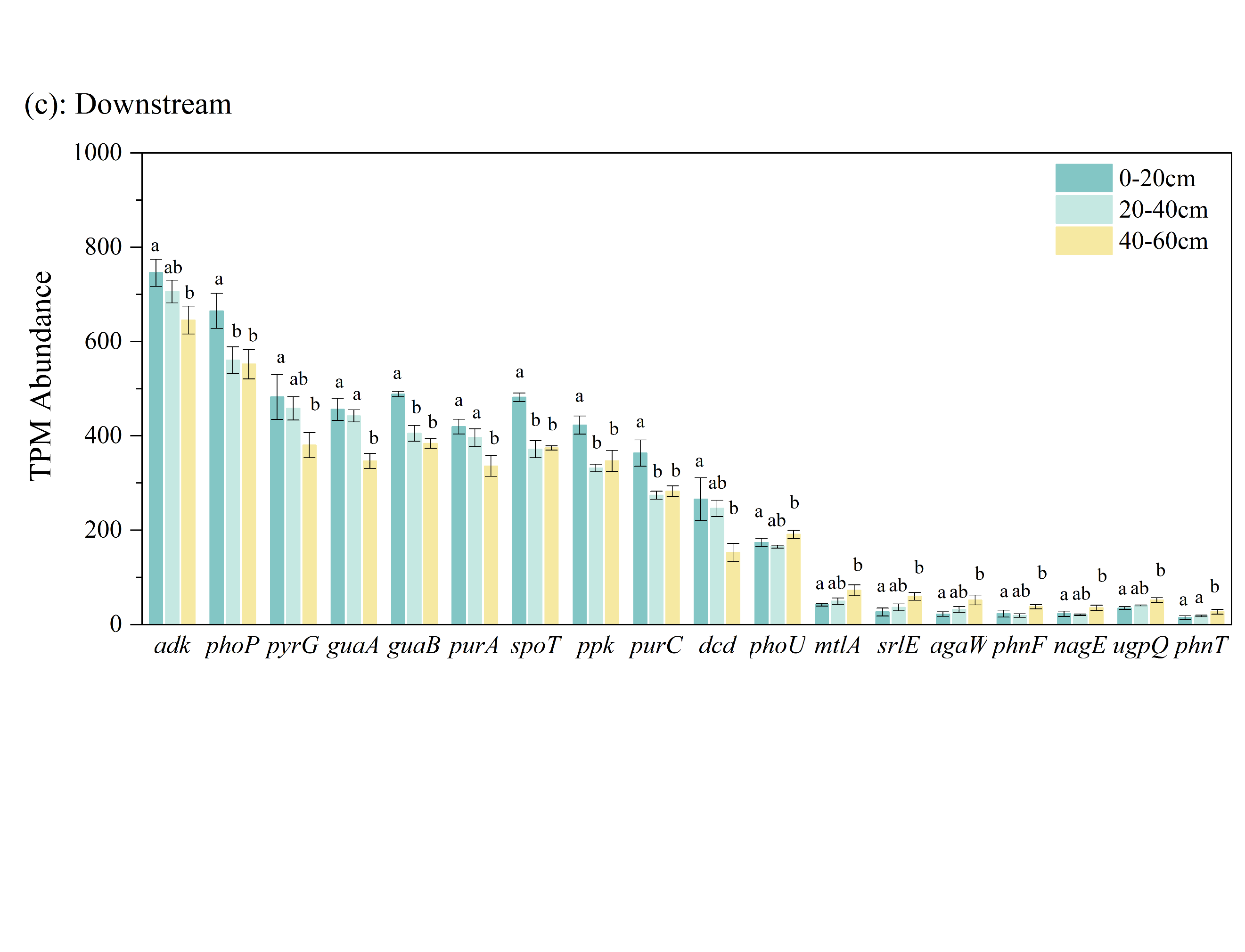
**

**Figure S6.** Abundance of functional genes of phosphorus metabolism at different depths in the upstream (a), midstream (b), and downstream (c) riparian zones. Upstream: upstream riparian zone; Midstream: midstream riparian zone; Downstream: downstream riparian zone. 0-20 cm, 20-40 cm, 40-60 cm, and 60-80 cm represent soil samples from the corresponding depths. Different letters indicate significant differences, p < 0.05.


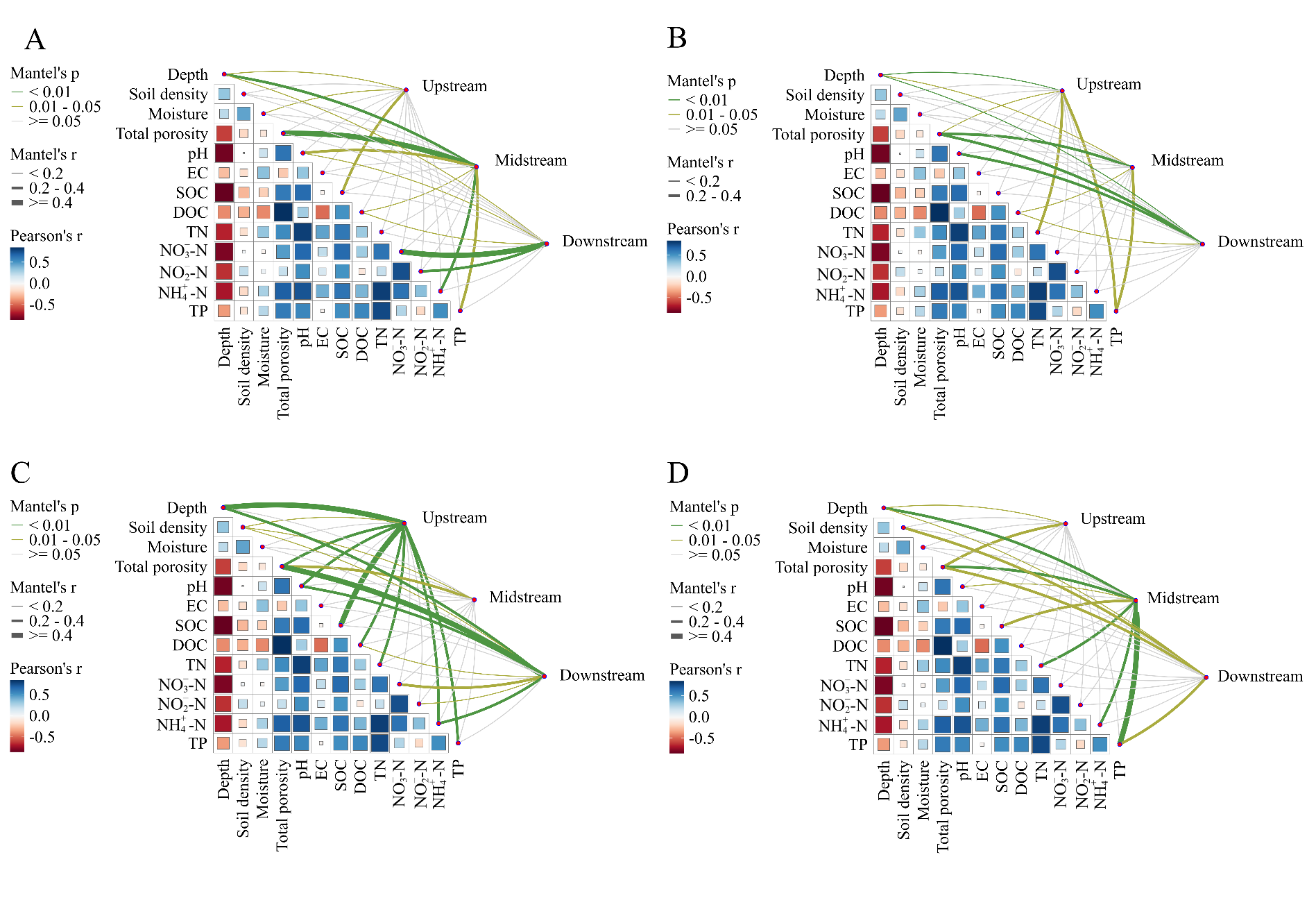


**Figure S7.** Correlation between microbial community structure (A) (determined by Bray-Curtis distance), carbon (B), nitrogen (C), and phosphorus (D) metabolism (determined by functional genes) with environmental factors, using the partial Mantel test. The partial Mantel’s r values are represented by edge width, and statistical significance is indicated by edge color. Pairwise correlations of environmental variables are shown using a color gradient reflecting the Spearman correlation coefficient. Upstream: upstream riparian zone; Midstream: midstream riparian zone; Downstream: downstream riparian zone.

## Supplementary Tables

**Table S1.** The distribution of samples by river section and soil depth

| **River Section** | **0–20 cm** | **20–40 cm** | **40–60 cm** | **60–80 cm** | **Total Samples** |
| --- | --- | --- | --- | --- | --- |
| Upstream | 3 | 3 | 3 | 3 | 12 |
| Midstream | 3 | 3 | 3 | 3 | 12 |
| Downstream | 3 | 3 | 3 | - | 9 |
| **Total** | **9** | **9** | **9** | **6** | **33** |

**Table S2.** Abundance of microbial carbon metabolism at different depths in the upstream, midstream, and downstream riparian zones.

|  | Upstream | Midstream | Downstream |
| --- | --- | --- | --- |
| Carbon decomposition | 5913.6±247.5 | 5966.132±988.2 | 5883.747±197.1 |
| Carbon fixation | 8441.8±384.1 | 7800.683±801.2 | 8413.498±372.4 |
| Methane metabolism | 3245.1±331.5 | 3299.816±586.5 | 3300.848±170.7 |
| Fermentation | 2476.9±284.3 | 2351.077±784.3 | 2456.976±112.2 |
| Aerobic respiration | 3144.6±474.9 | 3480.771±834.5 | 3206.871±66.6 |
| CO oxidation | 302.2±55.8 | 303.0164±38.6 | 250.2734±29.7 |

**Table S3.** Abundance of microbial nitrogen metabolism at different depths in the upstream, midstream, and downstream riparian zones.

|  | Upstream | Midstream | Downstream |
| --- | --- | --- | --- |
| Assimilatory nitrate reduction | 143.6±30.6 | 118.9±23.9 | 154.9±20.6 |
| Denitrification | 1060.2±130.3 | 1317.8±170.9 | 1004.4±109.1 |
| Dissimilatory nitrate reduction | 337.1±53.1 | 249.3±41.5 | 305.3±46.8 |
| Nitrification | 58.7±18.2 | 37.9±10.2 | 16.7±5.3 |
| Nitrogen fixation | 179.9±19.4 | 255.9±45.3 | 290.1±38.2 |
| Organic degradation and synthesis | 2470.3±222.9 | 2401.6±381.7 | 2462.2±107.3 |

**Table S4.** Abundance of microbial phosphorus metabolism at different depths in the upstream, midstream, and downstream riparian zones.

|  | Upstream | Midstream | Downstream |
| --- | --- | --- | --- |
| Organic phosphoester hydrolysis | 307.7±30.0 | 218.7±43.4 | 359.1±53.6 |
| Oxidative phosphorylation | 533.9±62.1 | 581.0±72.6 | 594.4±38.7 |
| Pentose phosphate pathway | 1104.5±118.1 | 940.2±77.5 | 951.3±78.4 |
| Phosphonate and phosphinate metabolism | 780.0±149.4 | 460.2±60.9 | 605.3±88.9 |
| Phosphotransferase system | 2689.7±332.1 | 1864.4±244.7 | 2251.2±179.3 |
| Purine metabolism | 5565.4±561.6 | 5998.8±815.2 | 5749.3±288.3 |
| Pyrimidine metabolism | 3030.0±293.5 | 3011.3±592.9 | 2986.7±150.2 |
| Pyruvate metabolism | 987.4±104.6 | 1030.5±108.9 | 1143.7±59.5 |
| Transmembrane transport | 2111.5±369.3 | 1552.4±215.2 | 1857.2±149.2 |
| Two-component system | 2626.8±256.6 | 2368.3±320.9 | 2553.7±308.5 |
